# Novel Azole-Modified Porphyrins for Mitochondria-Targeted Photodynamic Therapy

**DOI:** 10.3390/molecules30132688

**Published:** 2025-06-21

**Authors:** Sabarinathan Rangasamy, Elisa Bandini, Alessandro Venturini, Giuseppina Bozzuto, Sofia Migani, Annarica Calcabrini, Simona Sennato, Caterina Zuffa, Lucia Maini, Anaïs Brion, Frédéric Bolze, Cecilia Bombelli, Barbara Ventura

**Affiliations:** 1 Institute for Organic Synthesis and Photoreactivity (ISOF), National Research Council of Italy (CNR), Via P. Gobetti 101, I-40129 Bologna, Italy; elisa.bandini@isof.cnr.it (E.B.); alessandro.venturini@isof.cnr.it (A.V.); 2 Department of Chemistry, PSG College of Technology, Avinashi Rd, Peelamedu, Coimbatore IN-641004, Tamil Nadu, India; 3 National Centre for Drug Research and Evaluation, Italian National Institute of Health, Viale Regina Elena 299, I-00161 Rome, Italy; giuseppina.bozzuto@iss.it (G.B.); sofia.migani@iss.it (S.M.); annarica.calcabrini@iss.it (A.C.); 4 School of Science and Technology, Chemistry Division, University of Camerino, via Madonna delle Carceri (ChIP), I-62032 Camerino, Italy; 5 Institute for Complex Systems (ISC), National Research Council of Italy (CNR) and Physics Department, Sapienza University of Rome, Piazzale A. Moro 5, I-00185 Rome, Italy; simona.sennato@cnr.it; 6 Department of Chemistry “Giacomo Ciamician”, University of Bologna, Via F. Selmi 2, I-40126 Bologna, Italy; caterina.zuffa@unibo.it (C.Z.); l.maini@unibo.it (L.M.); 7 Laboratory of Synthetic and Therapeutic Chemo-Biology, UMR 7199, University of Strasbourg/CNRS, Faculty of Pharmacy, F-67401 Illkirch, Cedex, France; abrion@unistra.fr (A.B.); frederic.bolze@unistra.fr (F.B.); 8 Institute for Biological Systems (ISB), National Research Council of Italy (CNR), Secondary Office of Rome-Reaction Mechanisms c/o Department of Chemistry, Sapienza University of Rome, Piazzale A. Moro 5, I-00185 Rome, Italy; cecilia.bombelli@cnr.it

**Keywords:** azole, porphyrins, mitochondria, photodynamic therapy

## Abstract

Photodynamic therapy is a non-invasive treatment strategy for various types of cancer, based on the use of light to activate a photosensitizer which triggers processes leading to cell death. Given the increasing interest in the development of mitochondria-targeted photosensitizers, in this study we synthesized two novel thiadiazol-substituted porphyrins, 5,10,15,20-tetra(2,1,3-benzothiadiazol-5-yl) porphyrin (**C1**) and 5,10,15,20-tetra(1,2,3-thiadiazol-4-yl) porphyrin (**C2**), designed to target mitochondria in cancer cells thanks to the azole residues present in their structure. The two porphyrinic compounds were characterized in terms of structural and photophysical properties, revealing high yields of singlet oxygen production. Their interaction with biological structures was analyzed in a triple-negative human breast carcinoma cell line (MDA-MB-231), either as free compounds or delivered via mitochondriotropic liposome formulations. Both newly synthesized porphyrins entered MDA-MB-231 cells, with compound **C2** demonstrating more efficient localization in the cytoplasm and in mitochondria. Dark and phototoxicity tests were also performed: both compounds proved to be effective phototoxic agents, with **C2** showing the highest activity, making it a promising photosensitizer for mitochondria-targeted photodynamic therapy.

## 1. Introduction

Cancer is one of the deadliest diseases worldwide [1]. To alleviate its global impact and improve patient recovery rates, research on cancer treatment is indispensable. Furthermore, advancement in cancer research must address challenges such as side effects and recurrences [2]. Photodynamic therapy (PDT) is a highly selective and non-invasive treatment modality that has been approved for the treatment of esophageal, lung, and certain skin cancers [3,4,5]. The mechanism of action of PDT involves the activation of a photosensitizer (PS) with light of a specific wavelength, followed by the transfer of energy or electrons from the lowest triplet excited state of the PS to molecular oxygen or biological substrates. This process generates highly toxic reactive oxygen species (ROS), primarily singlet oxygen (^1^O_2_). These species are short-lived but highly destructive, inducing cellular damage and cell death through apoptosis, necrosis, and ferroptosis [6,7,8].

The properties of a PS are essential for effective PDT. An ideal PS should (a) absorb light in the red and far-red spectral region; (b) show minimal dark toxicity; (c) exhibit intense absorption bands within the irradiation range (molar absorption coefficient > 20,000–30,000 M^−1^ cm^−1^) to reduce the therapeutic dose; (d) produce ^1^O_2_ in high yields; (e) be fluorescent to monitor its subcellular localization [9,10,11]. Porphyrins and related macrocycles are extensively studied as PSs due to their ability to absorb light in the red/near-infrared region and efficiently generate singlet oxygen [12,13,14]. Low dark toxicity, high tumor specificity, and good biocompatibility make them suitable for PDT.

Photosensitizers are categorized into first, second, and third generations based on therapeutic needs [9]. Photofrin^®^, a first-generation PS, has long been the gold standard in PDT, despite its limitations. Its low molar absorption coefficient in the red region (1170 M^−1^ cm^−1^ at 630 nm), poor excretion, and low tumor selectivity resulted in long-lasting cutaneous photosensitivity in patients [15]. Because of these issues, a second generation of PSs was developed by modifying the porphyrin core to improve light absorption at longer wavelengths, enhance tumor specificity, and reduce skin photosensitivity. Recent advancements in targeting strategies have focused on achieving the selective accumulation of PSs in tumors, thereby minimizing damage to healthy tissues. These efforts have led to the development of third-generation PSs (targeted PDT) [16]. This approach acts specifically on molecular components associated with cancer [17,18], making it crucial to identify appropriate targets involved in cancer cell growth and survival.

In this context, mitochondria are potential therapeutic targets since their dysregulated function is responsible for tumorigenesis, tumor development, and tumor metastasis [19]. Mitochondrial DNA (mtDNA) is also directly involved in carcinogenesis due its high susceptibility to mutation and limited repair mechanisms [20,21]. Additionally, mtDNA is a target of ROS generated during mitochondrial respiration. Also, mitochondria are the main site for calcium accumulation, which is crucial for intracellular Ca^2+^ signaling, cell metabolism, and cell survival. Increased mitochondrial Ca^2+^ can trigger programmed cell death [22,23].

Interestingly, cancer cells exhibit unique mitochondrial properties that differ from non-malignant cells, such as producing ATP primarily through glycolysis—a phenomenon termed ‘aerobic glycolysis’ by Otto Warburg [24,25]. Some studies, including one by Simonnet et al., have demonstrated a correlation between glycolytic ATP production and tumor cell aggressiveness [26]. Moreover, cancer cells intelligently employ various mechanisms to evade the immune system within the tumor microenvironment. A recent finding by Yosuke Togashi revealed that cancer cells transfer defective mitochondria to immune cells, thereby weakening the body’s defenses and facilitating tumor evasion [27]. This process creates a favorable microenvironment for tumor progression.

Following the approach of mitochondria-targeted PDT [28,29,30], we designed novel porphyrins capable of selectively accumulating in mitochondria and acting as efficient photosensitizers. This goal was achieved by introducing azole groups into the porphyrin core structure. Azole groups are nitrogen-containing heterocyclic ring systems which may also contain additional heteroatoms such as sulfur and/or oxygen, and they are considered important for the development of new therapeutic agents [31,32]. Several azole compounds have been investigated for their potential to treat diseases that result from mitochondrial dysfunction, including cancer and neurodegenerative diseases [33,34,35]. Moreover, previous studies have shown that azoles can act on mitochondria by inhibiting mitochondrial respiration, modulating mitochondrial membrane potential, or interacting directly with mitochondrial enzymes or proteins. These properties make azoles promising anticancer agents, capable of inducing cancer cell death by inhibiting mitochondrial respiration and disrupting mitochondrial membrane potential, leading to release of pro-apoptotic factors [36,37].

In this work, two novel porphyrinic compounds containing 2,1,3-benzothiadiazole and 1,2,3-thiadiazole groups at the *meso* positions of the porphyrin core were synthesized and structurally characterized. Their absorption and emission properties, as well as singlet oxygen quantum yields, were evaluated in three different solvents, and their absorption features have been interpreted through a comprehensive theoretical study. The cellular uptake and localization of these compounds were investigated in a triple-negative human breast carcinoma cell line (MDA-MB-231). Cellular internalization studies revealed the ability of these molecules to penetrate cancer cells and specifically target mitochondria. Subsequently, PDT studies were conducted to evaluate their potential as phototoxic agents.

## 2. Results and Discussion

Conventional cancer therapies often cause significant side effects. Therefore, precise targeting of cancer cells and selective delivery of anticancer drugs are crucial for effective treatment. Through molecular design, PSs can be optimized for subcellular targeting and enhanced phototoxic activity. In our study, azole-substituted porphyrin macrocycles were designed to selectively localize in mitochondria and to act as effective PSs in PDT. Specifically, 2,1,3-benzothiadiazole-5-carbaldehyde and 1,2,3-thiadiazole-4-carbaldehyde were used to synthesize the novel porphyrins **C1** and **C2**, respectively.

### 2.1. Synthetic Procedure and Structural Characterization

#### 2.1.1. Synthesis

**Scheme 1:**
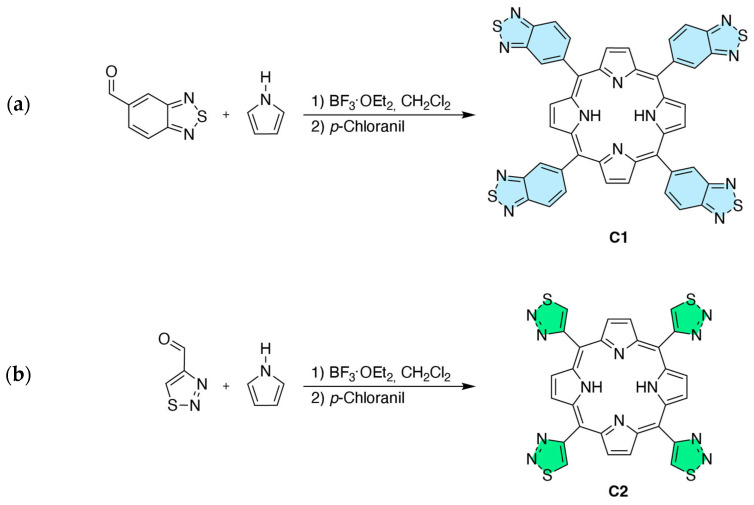
Synthesis of (**a**) 5,10,15,20-tetra(2,1,3-benzothiadiazol-5-yl) porphyrin **C1** and (**b**) 5,10,15,20-tetra(1,2,3-thiadiazol-4-yl) porphyrin **C2**.

The detailed procedure for the synthesis of **C1** and **C2** is provided in the Materials and Methods section. Briefly, **C1** and **C2** were synthesized using the Lindsey method [38,39], as shown in Scheme 1. This method is a well-known procedure for the preparation of *meso-*substituted and highly symmetric porphyrins, consisting of a two-step, one-flask synthesis performed at room temperature. **C1** and **C2** were synthesized via condensation of equimolar amounts of pyrrole with 2,1,3-benzothiadiazole-5-carbaldehyde and 1,2,3-thiadiazole-4-carbaldehyde, respectively, in the presence of a Lewis acid catalyst. Upon purification, the obtained yields were 37% for **C1** and 17% for **C2**.

#### 2.1.2. NMR Analysis

The NMR characterization of compounds **C1** and **C2** was performed by means of the ^1^H, ^13^C, COSY, and HSQC techniques. Detailed peak assignments are provided in the Supplementary Materials (Figures S1–S7).

The ^1^H NMR spectrum of **C1** in CDCl_3_ exhibits a characteristic porphyrin peak at −2.59 ppm, corresponding to highly shielded inner pyrrolic -NH protons, and a peak at 8.92 ppm, corresponding to *β*-pyrrole protons (H*_β_*), indicating the formation of a porphyrin framework (Figure S1). Additional peaks at 8.85 ppm (H6), 8.54 ppm (H8), and 8.34 ppm (H9) correspond to the 2,1,3-benzothiadiazole moiety at the *meso*-position of the porphyrin ring, as confirmed by the COSY experiment (Figure S2). The ^13^C NMR spectrum reveals carbon signals of the benzothiadiazole moiety at 154.19 ppm (C2), 154.56 ppm (C3), and 143.11 ppm (C7) (Figure S4). The HSQC spectrum (Figure S3) further confirms proton–carbon correlations with assignments at 126.34 ppm (C6), 136.12 ppm (C8), and 118.87 ppm (C9). Pyrrolic *α*- and *β*-carbons (C*_α_* and C*_β_*) are assigned considering the “NH tautomerism”, a well-known phenomenon in free-base porphyrins [40,41]. In the ^13^C NMR spectrum of **C1**, the C*_β_* signal appears as a broad and weak peak at 131.20 ppm, while the C*_α_* is undetectable, likely due to further signal broadening. This broadening derives from the isotopic effect on the rate of NH tautomerism within the porphyrin molecule. In some cases, these effects are so pronounced that the signals become undetectable, ultimately reducing the effectiveness of a “carbon count” correlation for many porphyrins [42]. Finally, the C*_meso_* peak is assigned at 118.82 ppm, almost overlapping the signal of C7.

Similarly, the ^1^H NMR spectrum of **C2** in DMSO-*d_6_* exhibits peaks at −2.85 ppm and 8.98 ppm, corresponding to the inner pyrrolic -NH protons and *β*-pyrrole protons (H*_β_*) of the porphyrin core, respectively. A peak at 10.24 ppm is related to the thiadiazole unit substituted at the *meso* position of the porphyrin ring (Figures S5 and S6). The ^13^C NMR spectrum assigns C5 and C1 at 161.48 and 142.64 ppm, respectively; C*_meso_* at 109.76 ppm; and C*_β_* at 132.27 ppm; while C*_α_* is undetected, as observed for **C1** (Figure S7).

#### 2.1.3. X-Ray Analysis 

**Figure 1:**
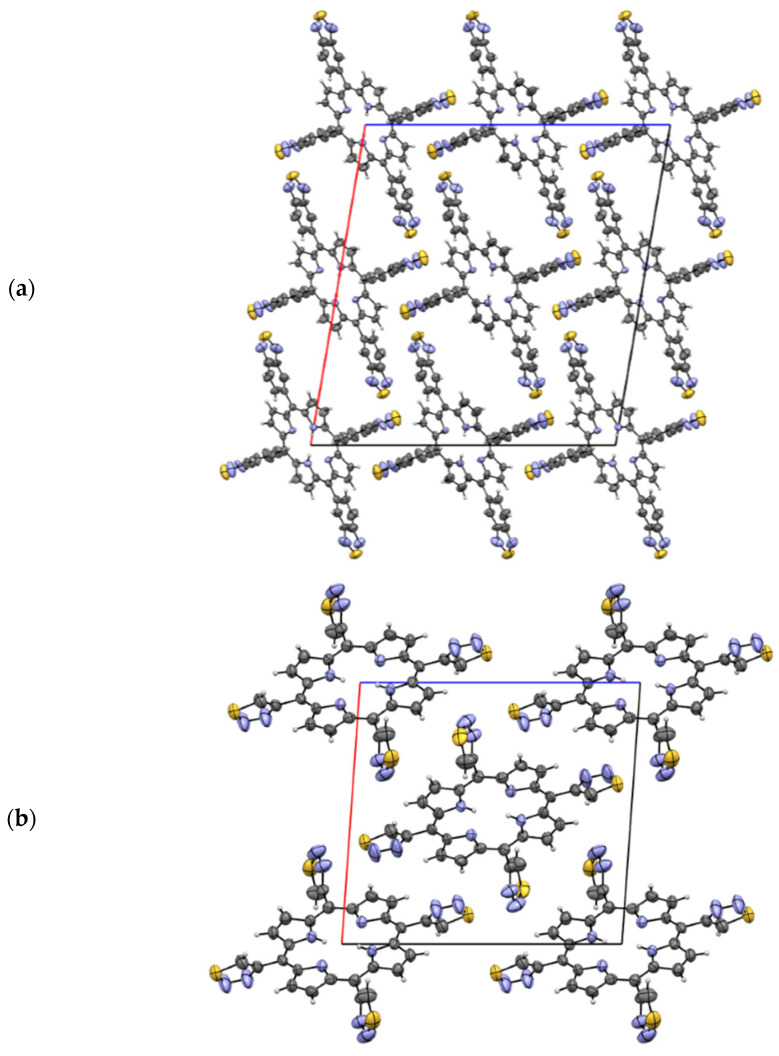
Crystal structure of (**a**) **C1** (disorder omitted for sake of clarity) and (**b**) **C2**. The packing view is along the *b*-axis in both cases. Cell axis color code: a is red, b is green, and c is blue. Atom color code: carbon is grey, nitrogen is blue, sulfur is yellow; ellipsoid at 50% of probability.

Single-crystal X-ray analyses of compounds **C1** and **C2** confirm their molecular structures as azole-substituted porphyrins (Figure 1).

Compounds **C1** and **C2** crystallize in the monoclinic system, with their molecules positioned on an inversion center, resulting in half a molecule in the asymmetric unit (Z’ = 0.5) with space group C2/c and P2/n, respectively. The porphyrin core of the molecule is planar, while the groups occupying the *meso* positions exhibit tilting relative to the porphyrin plane (detailed atom numbering is provided in Figure S8). In **C1**, the two benzothiadiazole moieties are inclined with respect to the porphyrinic core, forming dihedral angles (C18-C16-C17-C27 and C6-C7-C10-C11) of 61.07° and 79.75°, respectively. Notably, the first benzothiadiazole group adopts two distinct and opposing torsional angles, which causes the sulfur atom and one nitrogen atom to be disordered on two positions with 50% occupancy. The overall packing is characterized by the presence of empty channels that run parallel to the *b-*axis (Figure S8). In **C2**, the two thiadiazole moieties are tilted relatively to the porphyrinic plane, with dihedral angles (C1-C5-C6-C7 and C10-C12-C14-C15) of 73.29° and 64.52°, respectively. The molecules are linked through C-H∙∙∙N interactions (C8-H8∙∙∙N17 and C20-H20∙∙∙N3).

### 2.2. Photophysical Characterization

#### 2.2.1. Absorption and Emission Properties, Singlet Oxygen Production

The absorption and emission properties of **C1** and **C2** were studied in three solvents of different polarities (toluene, TOL; dichloromethane, DCM; and dimethyl sulfoxide, DMSO) using steady-state and time-resolved spectroscopic techniques.

**Figure 2:**
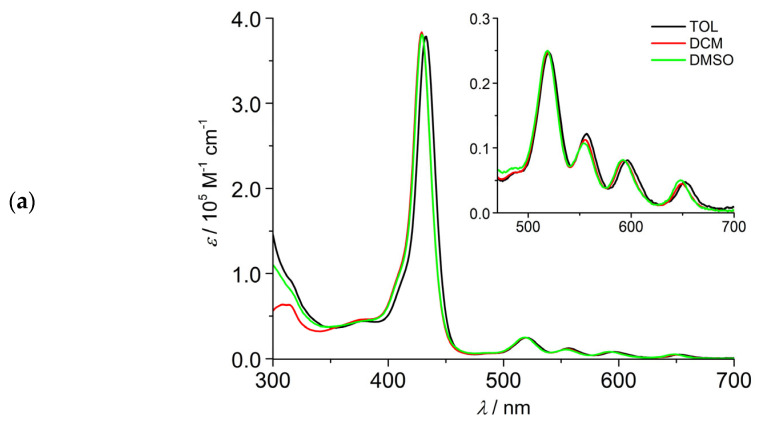

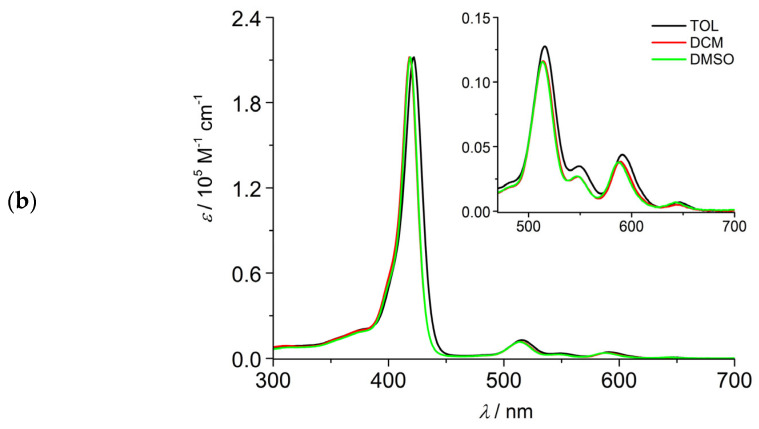
(**a**) Absorption spectra of **C1** in the three solvents (the spectrum in DMSO is arbitrarily scaled). (**b**) Absorption spectra of **C2** in the three solvents (the spectra in TOL and DMSO are arbitrarily scaled). Insets: magnification of the Q-band region (470–700 nm).

Figure 2 shows the absorption features of the two compounds in the three solvents, with arbitrarily scaled spectra when precise determination of the molar absorption coefficient (*ε*) was precluded by solubility reasons [43]. **C1** exhibits *ε* values (3.8 × 10^5^ M^−1^ cm^−1^ at the maximum of the Soret band in both TOL and DCM) and relative intensity of the bands typical of free-base porphyrins. However, the Soret band is largely red-shifted with respect to archetypal tetraphenylporphyrin (TPP), with a maximum at 433 nm in TOL (419 nm for TPP) [44], while the four Q-bands appear only slightly red-shifted (Figure 2a). Interestingly, the spectrum of **C2** shows a peculiar distribution of the Q-bands, and the *ε* of the Soret band in DCM (*ε*_418_ = 2.1 × 10^5^ M^−1^ cm^−1^) is significantly lower than that of **C1** (Figure 2b). On the other hand, the energy of the Soret transition in **C2** (maximum at 422 nm in TOL, Figure 2b) is similar to that of model TPP. The absorption features of the two compounds are not strongly affected by solvent polarity, exhibiting only a slight blue-shift of the bands when passing from TOL to DCM and DMSO. This shift is accounted by the polarizability of the solvent, indicating a *π*-π* nature of the involved transitions. A detailed computational analysis, revealing insights in the absorption properties of the two porphyrins, is reported below.

**Figure 3:**
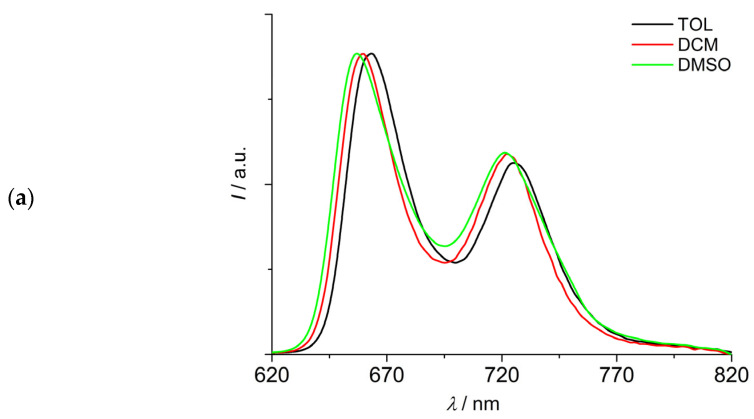

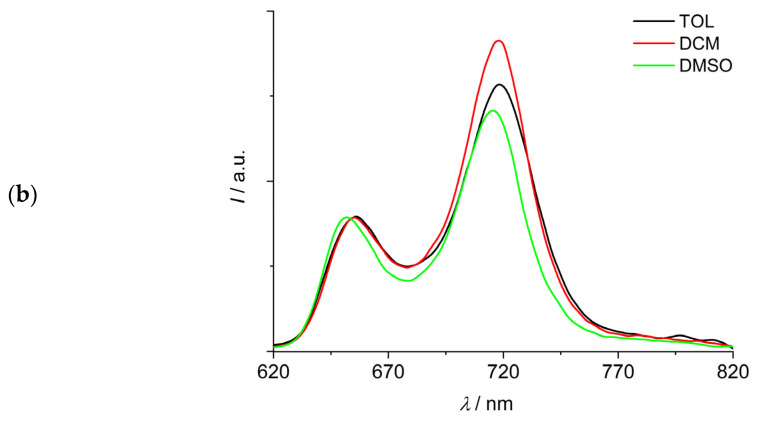
Normalized corrected emission spectra of (**a**) **C1** and (**b**) **C2** in the three explored solvents. *λ*_exc_ = 580 nm.

The emission properties of **C1** and **C2** were investigated in the same solvents. Figure 3 shows the emission spectra of the two compounds, while complete luminescence data are collected in Table 1. **C1** exhibits the typical fluorescence properties of free-base porphyrins in all solvents [44], with quantum yields between 0.11 and 0.14 and excited state lifetimes around 9–10 ns (Figure 3a and Table 1). Conversely, **C2** presents a particular behavior: together with a reversed relative intensity of the vibrational bands of the fluorescence spectrum (Figure 3b), its emission quantum yield and lifetime are considerably lower (ca. 5 and 3 times, respectively) than those of **C1** in all solvents. Analysis of radiative and non-radiative rate constants (Table 1) reveals both a reduction in the radiative channel and an enhancement in non-radiative deactivation pathways in **C2**, suggesting a geometry modification and/or activation of vibrational or rotational modes in the excited state.

The singlet oxygen quantum yield (*ϕ*_Δ_) is a critical parameter in determining the efficiency of a PS for PDT applications. It quantifies how effectively the PS converts the absorbed light into the production of a highly reactive oxygen species, i.e., singlet oxygen, which is a cytotoxic agent capable of inducing cancer cell death. Therefore, a detailed and accurate measurement of *ϕ*_Δ_ is essential for evaluating the PS characteristics. In this study, we measured the singlet oxygen production quantum yields of compounds **C1** and **C2** by using both direct and indirect methods, considering their solubility in the different solvents.

For **C1**, a direct method was employed in TOL and DCM: singlet oxygen phosphorescence at 1274 nm was collected upon excitation of the compound (and a suitable standard) at 442 nm (Figure S9). The measured yield was found to be similar in the two solvents, i.e., *ϕ*_Δ_ = 0.71 in TOL and *ϕ*_Δ_ = 0.75 in DCM (Table 1). These values are remarkably high and attest to the potential phototoxicity of compound **C1**. Due to the low solubility, the measurement in DMSO was excluded.

For **C2**, both direct and indirect methods were employed. In DCM, the direct collection of the singlet oxygen phosphorescence revealed a yield of *ϕ*_Δ_ = 0.73 (Figure S9b and Table 1), which is again a significant value for a porphyrin photosensitizer. The direct measurement in TOL was precluded by the low solubility of **C2** in this solvent. In DMSO, the use of an indirect method was required, due to the short lifetime of singlet oxygen in this solvent [45] that precludes direct phosphorescence determination. The procedure made use of 1,3-diphenylisobenzofuran (DPBF) as a singlet oxygen trap that is degraded by the presence of singlet oxygen. The rates of DPBF degradation for **C2** and the standard (Zinc phthalocyanine, ZnPc) were compared to calculate the singlet oxygen yield (see Figure S10 and the Materials and Methods Section for details), which was found to be very similar to that obtained in DCM, i.e., *ϕ*_Δ_ = 0.74 (Table 1).

Overall, the measured quantum yields of singlet oxygen generation for both compounds in all the explored solvents are in the range of 0.71–0.75 (Table 1), proving that the novel porphyrins **C1** and **C2** are efficient photosensitizers for PDT applications.

#### 2.2.2. Theoretical Modeling

In order to interpret the absorption spectral features of the two porphyrins **C1** and **C2**, we have carried out DFT and TD-DFT computational calculations. For both compounds, we have considered many structures with different symmetry, since the latter could affect the vibronic part of the systems. Moreover, because of the experimental solubility problems, for the theoretical analysis, we chose to use DCM, having an intermediate polarity between TOL and DMSO and being the solvent where the absorption spectra of both systems **C1** and **C2** could be precisely determined. In both cases, the more stable structures, indicated as **C1-C_i_** and **C2-C_i_**, were found to have an inversion center. Two other less (albeit slightly) stable structures with Cs symmetry were evaluated but are not discussed here (**C1-C_s_** and **C2-C_s_**, respectively, see Figure S11). The **C1-C_s_** isomer is less stable than **C1-C_i_** by 1.4 kcal/mol, and the **C2-C_s_** isomer is less stable than **C2-C_i_** by 1.2 kcal/mol.

All the systems exhibit a well-known absorption spectrum with characteristic Soret and Q bands: two couples of quasi-degenerate states give rise to the B (intense, Soret band) and two Q bands (dark), with the latter having a small value of *ε* and *f.* Each of these bands is actually composed of a couple of transitions oriented in the plane of the molecule. Figure S12 shows, for comparison, the two absorption spectra corresponding to the values of Table S2. Due to the well-known blue-shift effect of the type of functional used, the calculated spectra were red-shifted by 45 nm. Figure S13 shows the involved orbitals, as linear combinations, in the first four transitions, the two Q and the two Soret transitions, with coefficients close to 0.5 in **C1-C_i_**. In general, **C2** has an *ε* value smaller than **C1**, in agreement with the experimental outcome.

The Q bands are very weak, and a proper calculation of their spectra must take into account the FCHT effect. Figure S14 shows the results of the calculations of the vibronic spectra in the case of the two more stable isomers **C1-C_i_** and **C2-C_i_**. The calculated Q bands show differences between the two molecules, as experimentally observed. In **C2-C_i_,** the third band, starting from the lowest energy band, has a much smaller *ε* than in **C1-C_i_**. In other words, the vibronic spectrum of **C2-C_i_** is dominated by the two 0-0 transitions from the ground state to the zero-point energy of the first two excited states. Conversely, in **C1-C_i_**, the bands 91^1^ and 93^1^ are also intense (Figure S14a). The former corresponds to symmetric movement outside the plane of the central part of the porphyrin, while the latter derives from movement that also involves benzothiadiazol groups (see Figure S15).

### 2.3. In Vitro Studies

#### 2.3.1. Cellular Internalization

Intracellular uptake studies are crucial for understanding drug bioactivity, since they provide insights into the internalization and accumulation of the therapeutic agents within cancer cells. In this study, human breast adenocarcinoma MDA-MB-231 cells were utilized to analyze cellular internalization, as numerous studies suggest that PDT could serve as a new approach for the treatment and diagnosis of breast cancer, providing an alternative to conventional treatment modalities such as surgery, chemotherapy, and radiotherapy [46].

To test cellular internalization of the two newly synthesized PSs, and in view of their potential use in therapeutic protocols, they must be formulated to be soluble in biological fluids while maintaining, or improving, their performance. To this purpose, **C1** and **C2** were administered to cells in two different media: DMSO and a mixture of biocompatible solvents (solution F) already used in the pharmaceutical formulation of Foscan®.

We also studied the administration of the PSs vehicled by a liposomal formulation that could improve PS stability and targeting ability. We chose to include the two PSs into mitochondriotropic liposomes because this strategy has already been successfully explored to improve PDT in cancer treatment [47].

Liposomes were prepared with a natural unsaturated phospholipid (DOPC) and the triphenylphosphonium bolaamphiphile TPP3 [48,49], which has been shown to confer mitochondriotropic features to liposomes [50]. They were characterized from a chemical–physical point of view in terms of mean hydrodynamic size, polydispersity index (PDI), ζ-potential, and PS entrapment efficiency (EE%) (Table 2). Monodisperse vesicles with a hydrodynamic radius in the range of 50–60 nm, PDI of the order of 0.1, and ζ potential around 10 mV have been obtained. The mean diameter of the vesicles is in agreement with the expected size to be obtained by extrusion using membrane with pores of 100 nm; the radius, PDI, and ζ potential values did not change significantly after 8 days. The EE% of **C1** and **C2** in liposomes was 40% and 18%, respectively.

**Figure 4:**
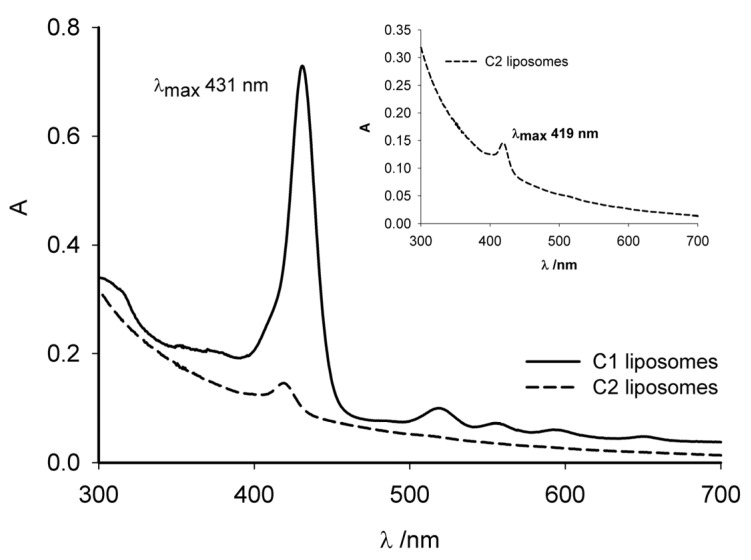
Absorption spectra of **C1** (solid line) and **C2** (dashed line) embedded in liposomes, obtained through diluting the samples at 1mM lipid concentration. Inset: enlargement of **C2** spectrum.

To evaluate the influence of the liposome environment on the absorption properties of **C1** and **C2**, the absorption spectra of the two PS-loaded liposome solutions were recorded (Figure 4). The samples were diluted to a proper lipid concentration at which the contribution of the scattering of liposomes to the absorption spectrum was reduced but the absorption peak of the PS was still clearly observable, i.e., 1 mM (the optical density of liposomes as a function of lipid concentration is reported in Figure S16). In these conditions, the concentration of **C1** is 1.8 μM (EE% 44) and that of **C2** is 0.7 μM, significantly lower because of its lower EE% (18%). It can be observed that the Soret band of **C1** is blue-shifted by only 2 nm with respect to organic solvents; moreover, the band is narrow and has no splitting, suggesting that the PS is in a monomeric form within the bilayer. For **C2**, the intensity of the Soret band is too low to derive spectral information, but the position of the peak reflects that observed in organic solvents.

#### 2.3.2. Intracellular Uptake of **C1** and **C2**

The intracellular uptake of **C1** and **C2** was assessed in MDA-MB-231 breast cancer cells using flow cytometry. This technique offers the advantage of measuring fluorescence from individual cells, enabling precise and quantitative evaluation of the cellular uptake, entrapment, and accumulation of fluorescent molecules, comparing samples from untreated versus treated conditions.

To perform cell treatments, and in order to establish the best conditions for the novel PSs to be administered to cells, compounds were dissolved either in DMSO or in the pharmaceutical solution F (40% ethanol and 60% propylene glycol). In the same experimental set, **C1** and **C2** were also administered as embedded in the mitochondriotropic liposomes. Then, 24 h after seeding, cells were treated with the PSs, either free or entrapped into liposomal formulations, for 4 and 18 h. Post treatment, the intracellular increase in fluorescence was quantified as the ratio between the mean fluorescence channel (MFC) in treated samples and that of untreated controls (CTRs). Conventionally, in flow cytometry, an increase in fluorescence intensity is indicative of compound uptake. The results indicate that **C2** exhibits better intracellular accumulation compared to **C1**, for both 4 and 18 h of treatment, when administered as dissolved in DMSO (Figure S17a). Similarly, when the PSs are dissolved in the pharmaceutical formulation (F), **C2** shows a higher fluorescence signal in comparison to **C1** (Figure S17b). When the PSs are encapsulated in liposomes, an increase in the fluorescence signal in the treated cells is also observed (Figure S17a,b). However, the signals are weaker than those revealed in cells treated with free PSs, at both times of treatment, suggesting different internalization kinetics.

#### 2.3.3. Intracellular Localization of **C1** and **C2**

The interaction of the novel porphyrin derivatives with cellular structures was studied by laser scanning confocal microscopy (LSCM) in order to evaluate their intracellular distribution in cancer cells. Firstly, we analyzed the intracellular localization of free **C1** and **C2** administered either in pharmaceutical solution F or in DMSO, in order to compare the effect of the two different biocompatible solvents in the mechanism of interaction of the novel PSs with biological structures. In the confocal images, the red signals represent the fluorescence of the molecules excited at 543 nm, while the green signals arise from mitochondria specifically labeled with 400 nM Mitotracker green. To finely visualize the co-localization of the PSs with the mitochondria (yellow signals) in the images, we reported optical sections of 1 nm thickness.

As visualized in Figure S18, in agreement with flow cytometry data, after 4 h of treatment, a little amount of **C1** (red signal) administered in pharmaceutical solution enters into MDA-MB-231 cells in the cytoplasm region, where labeled mitochondria are localized (green signal). After 18 h, **C1** aggregates inside the cytoplasm (red spots, arrows). The absence of yellow signals suggests that, in our experimental conditions, **C1** does not localize into mitochondria. **C2** administered in pharmaceutical solution is internalized more efficiently than **C1** by the cells. As shown in Figure S19, **C2** enters the cytoplasmic region of MDA-MB-231 cells and interacts with mitochondria, as revealed by yellow signals (arrows) in the merged image. In cells treated up to 18 h, **C2** red fluorescent spots finely distributed inside the cytoplasm (arrow heads) are visible. In addition, yellow regions in mitochondria suggest the fusion of **C2** with organelle membranes.

**Figure 5:**
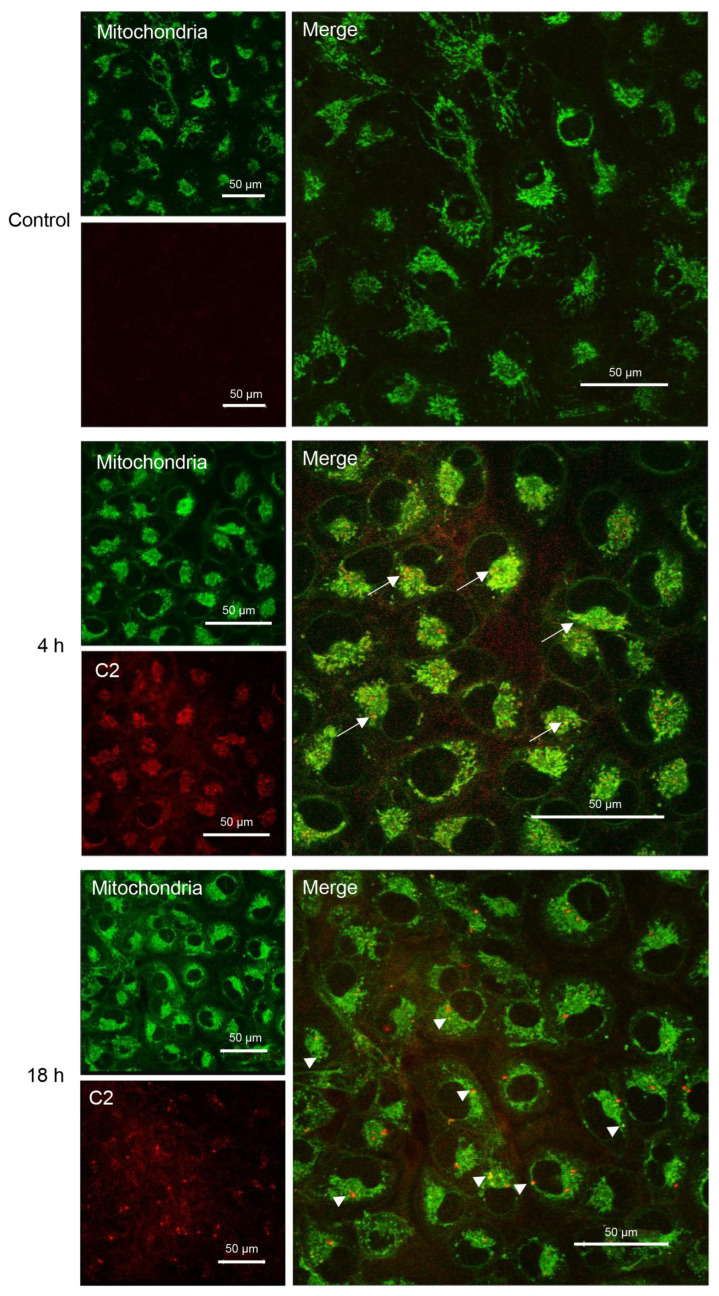
Intracellular localization of **C2** administered in DMSO to MDA-MB-231 cells for 4 and 18 h. Analysis by LSCM: optical sections. Green signal (488 nm): mitochondria; red signal (543 nm): **C2**. The PS localizes in the cytoplasmic region after 4 h treatment, as clearly visible in the images generated from only the red signal. After 18 h, **C2** begins to aggregate (red spots). In the enlarged merged picture, **C2** interacting with mitochondria (yellow spots, arrows and arrow heads) is clearly detected. In untreated cells (Control), no signal is revealed after excitation at 543 nm.

Analysis by LSCM was also performed on cells treated with the PSs dissolved in DMSO. The intracellular localization of **C1** appears to be similar to that of the same compound administered in pharmaceutical solution F (Figure S18). In contrast, accordingly with flow cytometry data, the presence of DMSO significantly improves the uptake of **C2** by MDA-MB-231 cells (Figure 5). Controls (untreated cells) exhibit an elongated, tubular mitochondrial morphology typical of healthy mitochondrial networks. Following 4 h treatment, a significant alteration in mitochondrial morphology is yet observed, characterized by a transition to a rounded and less defined structure. Under these experimental conditions, **C2** localizes in the cytoplasmic region, as clearly visible in the images generated from only the red signal, and after 18 h, it begins to aggregate (red spots). In the enlarged merged picture, it is also possible to see **C2** interacting with mitochondria (yellow spots, arrow heads).

All these observations clearly indicate that, in our experimental conditions, **C2**, differently from **C1**, interacts with mitochondria and that the dispersant medium influences the uptake of the compound; in particular, DMSO improves cellular uptake.

**Figure 6:**
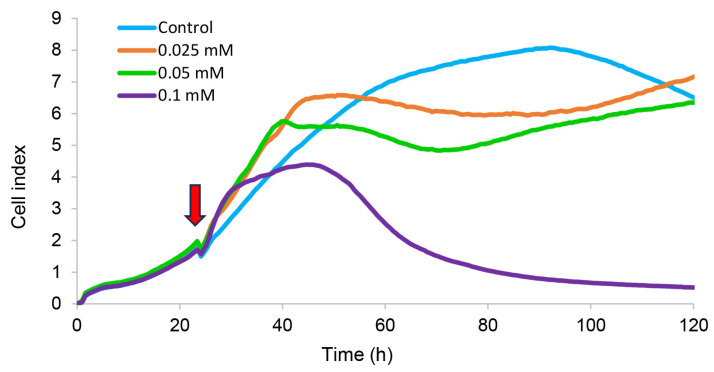
Analysis of DOPC/TPP3 liposome cytotoxicity by electrical impedance assay. Cell index of MDA-MB-231 cells continuously monitored by xCELLigence RTCA DP system is reported. The arrow indicates the addition of DOPC/TPP3 liposomes (0.025–0.05–0.1 mM) to the cell monolayer.

We further explored the biological properties of **C1** and **C2** loaded in mitochondriotropic liposomes, in order to verify if encapsulation was able to improve their interaction with intracellular structures. Given that mitochondriotropic liposomes are known to exhibit intrinsic cytotoxicity due to their mitochondrial targeting, we first assessed their cytotoxic effects [53,54]. This evaluation was conducted using a label-free, real-time cell analysis system (xCELLigence) that employs impedance-based technology to continuously monitor key cellular parameters, including viability, proliferation, morphology, adhesion, and migration. The system provides kinetic, non-invasive data without the use of dyes or terminal assays, offering a significant methodological advantage over traditional end-point techniques. The results are expressed in a unitless parameter called the Cell Index (CI), which reflects changes in electrical impedance as cells interact with sensor-covered microelectrodes [55]. The analysis performed on MDA-MB-231 cells grown for 120 h revealed that empty DOPC/Bola liposomes can be considered biocompatible at lower concentrations (0.025 and 0.05 mM), as they induce only a transient cytostatic effect (Figure 6). Cytotoxicity was observed only at the highest concentration (0.1 mM) from 24 h treatment onwards, indicating a time- and concentration-dependent effect on cell viability.

**Figure 7:**
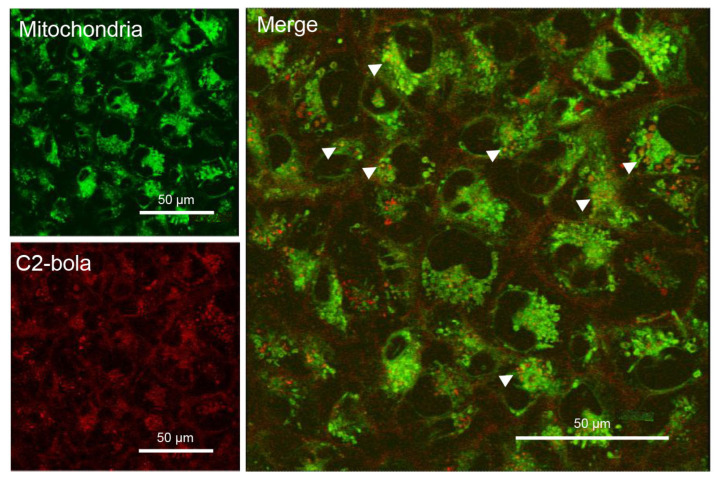
Intracellular localization of **C2** loaded in DOPC/TPP3 liposomal formulations (C2-bola) administered to MDA-MB-231 cells at a final lipid concentration of 0.1 mM for 4 h. Analysis by LSCM: optical sections. Green signal (488 nm): mitochondria; red signal (543 nm): C2-bola. Once entering the cells, C2-bola localizes inside the cytoplasm: the presence of orange-yellow spots in the merged image reveals the fusion of the porphyrin with mitochondria (arrow heads).

Based on these results, the cellular uptake and intracellular distribution of **C1** and **C2** delivered via DOPC/TPP3 liposomal formulations were subsequently analyzed by LSCM. To optimize the detection of the PS signal, the highest tested concentration (0.1 mM) was selected. Observations were performed 4 and 18 h after treatment, time points considered sufficient to study interaction and internalization dynamics, while still preceding the onset of detectable cytotoxicity. LSCM analysis revealed that, after 4 h, encapsulated **C1** enters the cells, but no interactions with mitochondria were observed, while **C2** carried by DOPC/TPP3 liposomes, once entering the cells, localizes inside the cytoplasm: the presence of orange-yellow spots reveals the fusion of the porphyrin with mitochondria (Figure 7, arrow heads).

It is noteworthy that liposomal **C2** enters the cells and co-localizes with mitochondria despite being trapped less efficiently than **C1**. Most likely, **C2**, due to lower EE%, perturbs less of the organization of the phospholipid bilayer, allowing the system as a whole to reach the mitochondrion thanks to functionalization with the bolaamphiphile TPP3. In contrast, it is reasonable to think that **C1**, which is encapsulated in the liposome bilayer in greater amounts than **C2** (see EE% data), modifies the characteristics of the liposome or destabilizes it, affecting its interaction with the biological membranes and intracellular fate.

Although liposomal formulation does not appear to increase photosensitizer uptake by the cells (see flow cytometry data), the result obtained with encapsulated **C2** is of interest. The presence of mitochondriotropic bolalipid in the liposomal formulation helps the interaction of PS-loaded vesicles with mitochondrial membranes and the release of the photosensitizer at the target site. In addition, it opens new possibilities for potential therapeutic applications since liposomes are still now among the nanovectors that are more employed in anticancer therapies [56]. Images of 18 h treated cells are not reported, since some toxicity of the PS–liposomal formulations was observed on cells seeded on the glass for the LSCM analysis (but not on treated cells seeded on tissue flasks for flow cytometry). The increased toxicity of liposomes in confocal microscopy experiments could be attributed to the different substrate on which the cells were grown to be observed by microscopy. In fact, it is known that the substrate influences cellular functions and the outcome of drug treatment in vitro [57].

Overall, the biological experiments evidence the capability of photosensitizers **C1** and **C2** to enter MDA-MB-231 human breast carcinoma cells. In our experimental conditions, both PSs were able to pass the cell membrane, with **C2** more efficiently localizing in the cytoplasm and then in the mitochondria of tumor cells.

#### 2.3.4. Dark Toxicity and Phototoxicity

**Figure 8:**
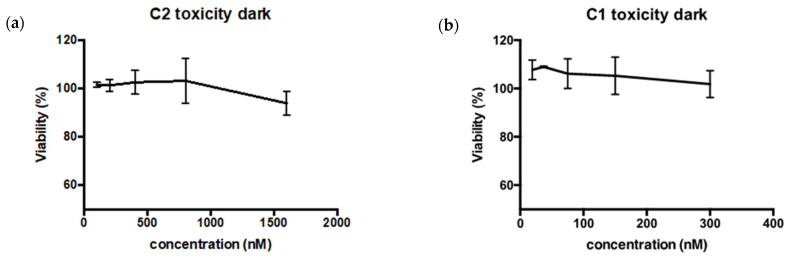
**C1** (**a**) and **C2** (**b**) dark cytotoxicity tests with MTS on MDA-MB-231 cells (after 24 h of incubation). The errors bars represent the standard deviation of the experimental values (technical triplicates and biological duplicates).

The dark toxicity of **C1** and **C2** was assessed using the MTS assay on MDA-MB-231 breast cancer cells. Figure 8a,b show the results obtained with increasing concentrations of **C1** and **C2**, respectively. For **C1**, concentrations up to 300 nM were tested, and cell viability remained above 90% after 24 h of incubation, indicating minimal to no dark toxicity. Similarly, **C2** was tested at concentrations up to 1600 nM, with cell viability also remaining around 90% after 24 h (the higher tested concentration for **C2** is related to its better solubility in DMSO). These results indicate that neither **C1** nor **C2** induces significant cytotoxicity under dark conditions.

**Figure 9:**
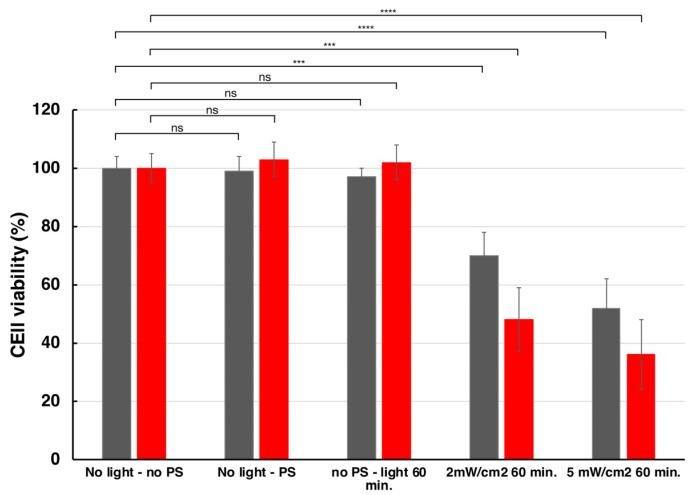
Phototoxicity of **C1** (300 nM, dark grey bars) and **C2** (1600 nM, red bars) on MDA-MB-231 cells upon 1 h irradiation at 515 nm (2 and 5 mW/cm^2^, 14 h drug-to-light delay, and toxicity test 24 h after irradiation). The errors bars represent the standard deviation of the experimental values (technical triplicates and biological duplicates). (ns: *p* > 0.05, ***: 0.0001 < *p* < 0.001 and ****: *p* < 0.0001).

Next, phototoxicity assays were performed on MDA-MB-231 cells using the optimized concentrations of **C1** and **C2** (300 and 1600 nM). Cells were seeded in a black 96-well plate (glass bottom) and incubated with **C1** (300 nM) and **C2** (1600 nM) for 14 h. The cells were then irradiated at 515 nm at the power of 2 mW/cm^2^ and 5 mW/cm^2^ for 1 h, as previously described [58]. Cell viability was assessed 24 h post irradiation using the MTS assay. As control experiments, the incubation with the PSs in the dark or irradiation without the PSs showed that the cell viability was not affected (Figure 9). On the contrary, upon irradiation, both **C1** and **C2** showed significant phototoxicity. Specifically, **C1** reduced the cell viability to 69% at 2 mW/cm^2^ and to 48% at 5 mW/cm^2^. In comparison, **C2** showed greater phototoxicity, reducing the viability to 53% at 2 mW/cm^2^ and 36% at 5 mW/cm^2^ (Figure 9). It is worth noting that both porphyrinic compounds are already effective as phototoxic agents at nM concentrations and that **C2** appears more promising since it has shown better cellular internalization and good mitochondrial co-localization. Moreover, the higher solubility of **C2** in DMSO and in culture media allows it to reach higher concentrations of the active PS, thus leading to increased phototoxic activity.

## 3. Materials and Methods

### 3.1. Synthesis

The aldehydes 2,1,3-benzothiadiazole-5-carbaldehyde and 1,2,3-thiadiazole-4-carbaldehyde (97% purity) were purchased from Fisher Scientific (Fisher Scientific Italia, Milan, Italy). Anhydrous dichloromethane (DCM, ≥99.8%), stabilized with 40–150 ppm amylene, boron trifluoride diethyl etherate (BF₃·OEt₂) and tetrachloro-1,4-benzoquinone (p-chloranil, 99%) were obtained from Merck Life Science srl, Milan, Italy. Pyrrole (for synthesis) was purchased from Merck Life Science srl, Milan, Italy, distilled slowly over calcium hydride prior to use, and stored at 4 °C in a brown bottle.

The ^1^H, ^13^C, COSY, and HSQC NMR spectra of porphyrins **C1** and **C2** were recorded using an Agilent DD2 500 MHz spectrometer equipped with a OneNMR probe. Chemical shifts (*δ*) are reported in ppm relative to residual solvent signals, and coupling constants (*J*) are given in Hz. Signal splitting patterns are abbreviated as follows: s = singlet; d = doublet; br s = broad singlet; br t = broad triplet; br q = broad quartet.

#### 3.1.1. Synthesis of 5,10,15,20-Tetra(2,1,3-benzothiadiazol-5-yl)porphyrin (**C1**)

In a thoroughly oven-dried 250 mL round-bottom flask, 2,1,3-benzothiadiazole-5-carbaldehyde (1 equiv, 1.83 mmol) was dissolved in anhydrous dichloromethane (DCM, 180 mL). Pyrrole (1 equiv, 1.83 mmol) was then added to the solution, and the mixture was stirred homogenously under inert conditions for 15 min. Subsequently, a catalytic amount of BF_3·_OEt_2_ (0.1 equiv, 0.18 mmol) was added dropwise using a syringe. The reaction mixture was stirred at room temperature under inert conditions for 3 h. After this, *p*-chloranil (0.5 equiv, 0.91 mmol) was added, and the reaction was exposed to air and refluxed for an additional 2 h. Upon completion, the solvent was removed under reduced pressure, and the crude product was purified by column chromatography on basic alumina. A red band eluted at 50:50 *v*/*v* DCM/hexane was identified as the target compound, **C1**. The purified compound (**C1**) collected as a purple solid was characterized using various analytical techniques. Yield: 37%. ^1^H NMR (500 MHz, CDCl_3_) *δ* ppm 8.92 (br s, 8H), 8.85 (br t, 4H), 8.54 (br q, 4H), 8.34 (d, *J* = 8.8 Hz, 4H), −2.59 (br s, 2H). ^13^C NMR (125 MHz, CDCl_3_) *δ* ppm 154.56, 154.19, 143.11, 136.12, 131.20, 126.34, 118.87, 118.82.

#### 3.1.2. Synthesis of 5,10,15,20-Tetra(1,2,3-thiadiazol-4-yl)porphyrin (**C2**)

In a thoroughly oven-dried 250 mL round-bottom flask, 1,2,3-thiadiazole-4-carbaldehyde (1 equiv, 2.62 mmol) was dissolved in anhydrous dichloromethane (DCM, 180 mL). Pyrrole (1 equiv, 2.62 mmol) was then added to the solution, and the mixture was stirred homogenously under inert conditions for 15 min. Then, a catalytic amount of BF_3·_OEt_2_ (0.1 equiv, 0.26 mmol) was added dropwise using a syringe. The reaction mixture was stirred at room temperature under inert conditions for 3 h. Finally, *p*-chloranil (0.5 equiv, 1.31 mmol) was added, and the reaction was exposed to air and refluxed for an additional 2 h. Upon completion, the solvent was removed under reduced pressure, and the crude product was purified using column chromatography on basic alumina. A red band eluted at 50:50 *v*/*v* DCM/hexane was identified as the target compound, **C2**. The purified **C2** compound collected as a purple solid was characterized using various analytical techniques. Yield: ~17%. ^1^H NMR (500 MHz, DMSO-d_6_) *δ* ppm 10.24 (s, 4H), 8.98 (br s, 8H), −2.85 (br s, 2H). ^13^C NMR (125 MHz, DMSO-d_6_) *δ* ppm 161.48, 142.64, 132.27, 109.76.

### 3.2. X-Ray Analysis

The single crystal data of **C1** and **C2** were collected at room temperature on an Oxford Xcalibur using Mo-ka radiation, equipped with a graphite monochromator and CCD Sapphire detector. The crystals were covered with inert oil (Fomblin YR-1800) and stuck on a suitable loop installed on a sample holder. Crystal data details are summarized in Table S1 in SI. The SHELXT [59] and SHELXL [60] algorithms were used for the solution and refinement of the structures based on F^2^. All the atoms, excluding the hydrogens, were refined anisotropically. Hydrogen atoms have been added to the theoretical positions. CCDC 2343048-2343049 contains the supplementary crystallographic data for this paper. The data can be obtained free of charge from The Cambridge Crystallographic Data Centre via www.ccdc.cam.ac.uk/structures. The graphical representations of the structures were displayed with the software MERCURY [61].

### 3.3. Spectroscopy and Photophysics

Spectroscopic-grade DMSO, DCM, and TOL were purchased from Merck. Rose Bengal bis(triethyl-ammonium) salt, DPBF, Zn-phthalocyanine (ZnPc), and *meso* tetraphenylporphyrin (TPP) were from Aldrich.

Absorption spectra were recorded with a PerkinElmer Lambda 950 UV-Vis-NIR spectrophotometer. Emission spectra were collected with an Edinburgh FLS920 fluorimeter, equipped with a Peltier-cooled Hamamatsu R928 PMT (290–850 nm), and corrected for the wavelength-dependent phototube response. Fluorescence quantum yields have been determined with reference to TPP in aerated TOL (*ϕ*_fl_ = 0.11) [62]. Fluorescence lifetimes have been measured with an IBH Time Correlated Single Photon Counting apparatus with nanoLED excitation at 560 nm. The analysis of the luminescence decay profiles against time was accomplished with DAS6 Decay Analysis Software provided by the manufacturer.

Singlet oxygen production quantum yields in DCM have been measured with reference to Rose Bengal bis(triethyl-ammonium)salt (*ϕ*_Δ_ = 0.48) [63], by comparing the intensity of singlet oxygen phosphorescence spectra measured with an FLS920 fluorimeter (Edinburgh) equipped with a Hamamatsu R5509-72 InP/InGaAs photomultiplier tube supercooled at 193 K in liquid nitrogen-cooled housing and a TM300 emission monochromator with NIR grating blazed at 1000 nm (sensitivity range: 300–1700 nm). Excitation at 442 nm has been performed with a Kimmon Koha Co. Ltd. (Tokyo, Japan). HeCd laser (with the power reduced to ca. 7 mW by using a neutral density filter to avoid annihilation effects). The same measurements in TOL have been performed, making use of TPP as a standard (*ϕ*_Δ_ = 0.70) [64].

Singlet oxygen production quantum yields in DMSO have been measured with an indirect method using DPBF as a singlet oxygen trap [58]. ZnPc has been used as a standard (*ϕ*_Δ_ = 0.67) [65]. Solutions of the standard (8.3 × 10^−6^ M) or the compound (ca. 9.0 × 10^−5^ M), containing DPBF 2.2 × 10^−5^ M, prepared in the dark, have been irradiated at 600 nm (*A*_600_ ≈ 0.2 for both compound and standard) by using an irradiation setup composed by a 150 W xenon lamp (LOT) and an Omni-λ150 monochromator (Zolix) completed by a 590 nm cutoff filter, under continuous stirring. The light intensity was 0.30 mW/cm^2^. The decrease in DPBF absorption as a function of the irradiation time, derived by subtracting the constant absorption contribution of the compound or the standard from the spectrum of the mixture at each irradiation point, has been followed both at 445 nm and 327 nm, with similar results, in order to avoid distortions given by the subtraction of the intense Soret band.

Estimated errors are 10% for exponential lifetimes, 20% for quantum yields, 20% for molar absorption coefficients, and 3 nm for emission and absorption peaks.

### 3.4. Theoretical Parameters

The calculations have been carried out using Gaussian 16 software [66]. The used methods were DFT and TD-DFT, the wB97XD long-range corrected hybrid functional including dispersion corrections [67], and the Def2SVPP basis-set [68]. To predict vibronic spectra, the combined Franck–Condon and Herzberg–Teller methods have been used as described in Gaussian 16 software at 0 K [69].

The absorption spectra calculated using the theoretical methods were red-shifted by 45 nm, as is usually performed with many DFT functionals containing long-range corrections to better compare them with experimental spectra [70]. The X, Y, and Z coordinates of ground-state optimized systems with C_i_ symmetry are reported at the end of the Supplementary Materials Section.

### 3.5. Cells Studies on Free and Liposome-Loaded PSs

#### 3.5.1. Materials

DMEM medium, penicillin/streptomycin solution (100X), L-glutamine solution at 200 mM, and MEM non-essential amino acids (100X) were purchased from EuroClone S.p.A. RPMI-1640 medium was purchased from Dutsher. Fetal bovine serum (FBS) was purchased from Hyclone, Carmlington, UK. DMSO and phosphate-buffered solution (PBS) were purchased from Sigma Aldrich. Propidium iodide (PI) was purchased from Applichem, and Mitotracker green was purchased from Molecular Probes. 1,2-dioleoyl-*sn*-glycero-3-phosphocholine (DOPC) (purity ≥ 99%) was purchased from Avanti Polar Lipids (Alabaster, AL, USA). Phosphate-buffered saline (PBS; 0.01 M phosphate buffer; 0.0027 M KCl; 0.137 M NaCl; pH 7.4), absolute ethanol (purity ≥ 95.0%), chloroform, and DCM were purchased from Sigma Aldrich. Propylene glycol, DMSO, DCM, analytical-grade RPE, and RS-grade CHCl_3_ were purchased from Carlo Erba reagents. Milli-Q water was produced with a Direct-Q3 Millipore apparatus and used for the preparation of the PBS buffer.

The TPP bolaamphiphile TPP3 was synthesized as previously described [48,49].

#### 3.5.2. Instrumentation

An LSRII flow cytometer (Becton and Dickinson, Franklin Lakes, NJ, USA) equipped with a 5 mW, 488 nm, air-cooled argon ion laser and a Kimmon HeCd 325 nm laser was used for the analysis of photosensitizer (PS) uptake.

A Leica TCS SP2 laser scanning confocal microscope (LSCM, Leica, Microsystems, Mannheim, Germany) equipped with an Ar/Kr laser was used to study the intracellular distribution of the PSs.

An xCELLigence RTCA (Real-Time Cell Analysis) DP (Dual Purpose) system (Agilent Technologies, Santa Clara, CA, USA), an impedance-based measuring device, was used to analyze cell cytotoxicity after liposomal formulation treatment.

A 10 mL Lipex Biomembranes extruder, equipped with Whatman Nuclepore polycarbonate membranes (pore size 0.1 μm), was used for liposome preparation.

A probe-tip sonicator (Sonics Vibra-Cell) and a rotary evaporator (Buchi Rotavapor R-210) were used in the preparation of liposomes and solutions containing **C1** and **C2**.

A Cary 300 UV-vis double beam spectrophotometer (Varian Australia PTY Ltd., Mulgrave, Vic., Australia), equipped with a thermostat apparatus for temperature control, was used to record UV-vis spectra of liposomes and PS-loaded liposomes and to evaluate the PSs’ EE%.

The NanoZetaSizer apparatus (Malvern Instruments, Worcestershire, United Kingdom), in backscattering configuration and equipped with a 5 mW HeNe laser (*λ* = 632.8 nm), was used to measure the size and the electrophoretic mobility of the samples. The cumulant method was applied to determine the hydrodynamic diameter and the olydispersity index (PDI) [71]. For the determination of the *ζ*-potential, the electrophoretic mobility (*μ*) was converted into *ζ*-potential by the Smoluchowski relation, *ζ* = *μ* *η*/*ε*, with *η* and *ε* the solvent viscosity and permittivity, respectively [72].

#### 3.5.3. Preparation of **C1** and **C2** Solutions for LSCM and Flow Cytometry

Solutions of **C1** and **C2** 3.7 × 10^−5^ M were prepared as follows:

**C1(DCM)**: a 0.25 mM stock solution of **C1** was prepared via weighing 0.85 mg (0.001 mmol) in a volumetric flask (4 mL) and filling it up to the mark with DCM.

**C1(DMSO)**: 150 μL of **C1(DCM)** was transferred in a 1 mL volumetric flask and the mark was filled with DMSO to obtain a 3.7 × 10^−5^ M solution.

**C1(F)**: First, 150 μL of **C1(DCM)** was transferred in a 1 mL volumetric flask, and the mark was filled with a propylene glycol (560 mg)/absolute ethanol (376 mg) solution, called solution F because it is the medium of the pharmaceutical preparation Foscan®. After dilution, the **C1** concentration was 3.7 × 10^−5^ M.

**C2(DMSOa)**. A 0.43 mM stock solution of **C2** was prepared weighting 1.4 mg (0.002 mmol) in a volumetric flask (5 mL) and filling it up to the mark with DMSO. This solution has been used to prepare **C2(F)**; see below.

**C2(DMSOb)**: A 3.7 × 10^−5^ M solution was prepared by weighting 1.2 mg (0.0018 mmol) of **C2** in a volumetric flask (50 mL) and filling it up to the mark with DMSO. This DMSO solution has been used to perform biological experiments.

**C2(F)**: 86 μL of 0.43 mM **C2(DMSOa)** was transferred in a 1 mL volumetric flask, and the mark was filled with solution F (40% ethanol and 60% propylene glycol), to obtain a 3.7 × 10^−5^ M solution.

Given the tendency of **C1** and **C2** to aggregate, all stock solutions were left in the dark overnight before use, under magnetic stirring.

#### 3.5.4. Preparation of Mitochondriotropic Liposomes

PBS buffer dispersions of mitochondriotropic liposomes, loaded with **C1** or **C2**, were prepared according to the thin-film hydration method, coupled with the *freeze–thaw* protocol, as described previously [73,74]. Briefly, proper amounts of DOPC (31.8 mM stock solution) and TPP3 (14.7 mM stock solution) chloroform solutions, as well as **C1** or **C2** DCM solutions (≈0.3 mM), were added in a round-bottom flask to obtain a mixture with a PS/total lipid molar ratio of 1:250 and a DOPC/TPP3 molar ratio of 9.75:0.25. By evaporation of the organic solvents, thin lipid films were obtained on the wall of the flasks and kept overnight under reduced pressure (0.4 mbar). The films were then hydrated with the proper amount of PBS to have final dispersions of 10 mM in total lipids and 0.04 mM in **C1** or **C2**. The dispersions were vortex-mixed in order to completely detach the film from the flask wall. The resulting multilamellar vesicles (MLVs) were freeze–thawed six times from liquid nitrogen to 50 °C and extruded (10 times) through a 100 nm polycarbonate membrane at 45 °C.

Absorption spectra of **C1** and **C2** embedded in liposomes were recorded upon dilution to 1 mM total lipids, in order to reduce liposome-associated scattering, highly present below 500 nm and thus in the spectral region of the Soret band.

#### 3.5.5. Determination of **C1** and **C2** Entrapment Efficacy (EE%)

The **C1** or **C2** concentration in liposomes before and after extrusion was determined by UV-vis spectroscopy measurements, after construction of the calibration curves for the two PSs.

#### 3.5.6. Calibration Curves

First, 1.1 mg of **C1** or **C2** was weighted in 50 mL volumetric flasks and the mark was filled with DCM or DMSO, respectively, obtaining 2.60 × 10^−5^ M **C1** and 3.65 × 10^−5^ M **C2** solutions. By successive dilutions, different solutions of the two PSs in the range of 0–3 × 10^−6^ M were obtained. The absorbance of each solution was measured at 25 °C, in quartz cuvettes of 1 cm path-length. By plotting the absorbance maximum (*λ*_max **C1**_ = 429 nm; *λ*_max **C2**_ = 419 nm) as a function of PS concentration and fitting the experimental data, the calibration curves for **C1** and **C2** were obtained.

#### 3.5.7. Determination of EE%

To disrupt the aggregates, 500 µL liposome suspensions were brought to dryness by high vacuum. The residues were suspended in 3 mL of DMSO (**C2**) or DCM (**C1**), and the maximum absorbance was measured; finally, the concentration was calculated by the calibration curve. The percentage of entrapped PS was calculated by Equation (1):

(1) $$EE\%=\frac{M_{aPS}}{M_{bPS}}*100$$

where *M_aPS_* and *M_bPS_* are the PS concentrations after and before liposome extrusion, respectively.

#### 3.5.8. Cell Cultures

MDA-MB-231 cells were obtained from American Type Culture Collection (ATCC CRM-HTB-26). They were cultured in DMEM medium (or RPMI-1640 medium for dark and phototoxicity experiments) and supplemented with 10% FBS, penicillin, and streptomycin (100 IU/mL/100 mg/mL) at 37 °C in a cell incubator at 95% relative humidity with 5% CO_2_ supply.

#### 3.5.9. Flow Cytometry

The analysis of PS uptake was performed in MDA-MB-231 cells treated with free PSs (3.7 × 10^−5^ M DMSO or F solutions) or encapsulated in liposome formulations for 4 and 18 h. Both the liposome dispersion and DMSO and F solutions were diluted 1:100 in culture medium for the treatment. At the end of each treatment, cells were washed with ice-cold PBS, detached with EDTA and trypsin, resuspended in ice-cold PBS, and immediately analyzed by flow cytometry for PS content. Fluorescence signals were analyzed on an LSRII flow cytometer. Fluorescence emissions were collected through a 570 (FL2) and 670 (FL3) nm band-pass filter in order to analyze propidium iodide (PI) and PS signals, respectively. At least 10,000 cells per sample were acquired in log mode. Only PI-negative (viable) cells were considered for analysis. The fluorescence intensity values were expressed as the mean fluorescence channel (MFC) using FACS Diva software (Becton Dickinson).

The ratios between the MFC of cells incubated with PS and that of control ones (cells treated with vehicle alone) were calculated to quantify the increase in fluorescence emission (related to PS uptake) with respect to the signal of autofluorescence derived from untreated samples (Diva software, v5.0.3).

#### 3.5.10. Electrical Impedance Assay

The cytotoxicity of mitochondriotropic liposomes was continuously monitored using the xCELLigence RTCA (Real-Time Cell Analysis) DP (Dual Purpose) system, an impedance-based measuring device. The xCELLigence station was housed in a standard incubator maintained at 37 °C with 5% CO₂ and was continuously connected to a computer equipped with RTCA Software Pro, version 2.2, via the RTCA DP control unit. Impedance measurements were recorded by applying an alternating voltage of 22 mV at a frequency of 10 Hz. For all experiments, E-Plate 16 microplates (Agilent) were used. Impedance sensors embedded in the bottom of each well automatically detected changes in electron flow caused by adherent cells. These changes were expressed as a unitless parameter called the Cell Index (CI), calculated as follows: CI = (impedance at time point n—impedance in the absence of cells)/nominal impedance value.

Briefly, MDA-MB-231 cell suspensions were seeded in an E-Plate 16 well (5 × 10^3^ cells/150 µL medium/well). The plate was left undisturbed at room temperature for 30 min to allow cells to settle and adhere and then placed into the RTCA DP station to initiate real-time impedance monitoring. Cells were allowed to grow and attach for approximately 24 h, until a stable monolayer was established, as indicated by a plateau in the Cell Index. The E-Plate 16 was carefully removed under sterile conditions to administer DOPC/Bola liposomes (0.025, 0.05, and 0.1 mM) and then returned to the RTCA DP station. Impedance readings were acquired every 30 min for up to 120 h post treatment.

#### 3.5.11. Laser Scanning Confocal Microscopy

In order to investigate the intracellular distribution of the free PSs or those carried by the DOPC/TPP3 liposome formulation, living MDA-MB-231 cells were analyzed by laser scanning confocal microscopy (LSCM). Cells (10^4^ cells/cm^2^) were seeded in WillCo Petri dishes and, after 24 h, incubated with 3.71 × 10^−5^ M free PSs or PSs loaded in liposomes (final concentration 0.1 mM). After incubation for 4 and 18 h at 37 °C, cells were incubated with Mitotracker green 400 nM for 10 min at 37 °C, fixed in 1% paraformaldehyde in PBS for 10 min at room temperature, and, finally, observed with the laser scanning confocal microscope.

#### 3.5.12. Dark Toxicity and Phototoxicity

For dark toxicity and phototoxicity experiments, cell viability was assessed using MTS assays. Briefly, cells were seeded in 96-well plates at a density of 2 × 10^4^ cells per well in 100 μL of RPMI-1640 medium. Then, 8 h after seeding (a suitable period for the cells to attach to the glass bottom and start to grow), cells were treated with the PS compound (stock solutions prepared in DMSO at a concentration of 38 and 160 μM for **C1** and **C2**, respectively, related to the maximum solubility of the two compounds), while cells without treatment were used as controls (viability = 100%).

For phototoxicity experiments, after 14 h drug-to-light delay (DLI), necessary to allow optimal cell penetration, the cells were irradiated (or not) with a home-made setup [58] for 1 h using an LED source at a power intensity of 2 or 5 mW.cm^−2^. The setup consisted of a homemade apparatus using twenty-four 5 mm LEDs (515 nm, 14,000 lm) (KingBright L-7113ZGC, Los Angeles, CA, USA) adapted for irradiating 24 independent wells of a Corning 96-well black special optic plate. Six groups of 4 LEDS soldered in series were connected in parallel, allowing the irradiation of these 24 wells. A variable laboratory power supply (BaseTech BT-305, Herndon, VA, USA) was used to power the LED array with fine control of voltage and intensity. The light power was measured for each well using a Thorlabs PM100D power meter.

After 24 h, an MTS cell viability assay was carried out by adding 20 μL of MTS (CellTiter 96^®^ AQ_ueous_ One Solution Cell Proliferation Assay) to each well followed by incubation at 37 °C for 30 min. Finally, the absorbance values of the full plate were measured at 490 nm using a microplate reader (Safas SP2000, Xenius 5801, Dubai, United Arab Emirates).

All in vitro experiments were conducted in technical triplicates and biological duplicates.

#### 3.5.13. Statistical Analysis

To assess the variability of the experimental measurements, the standard deviation was calculated for each set of data and reported as the error. In the phototoxicity experiments, an unpaired *t*-test was performed using GraphPad (ns: *p* > 0.05, ***: 0.0001 < *p* < 0.001 and ****: *p* < 0.0001).

## 4. Conclusions

Two novel thiadiazol-substituted porphyrins have been designed, synthesized, and characterized in view of their possible use as mitochondria-targeted photosensitizers. A detailed photophysical study, supported by theoretical investigations, evidenced peculiar absorption and emission properties and a remarkable capacity of both molecules to produce singlet oxygen. Interestingly, both compounds were found to internalize in human breast carcinoma MDA-MB-231 cells, with **C2** able to localize more efficiently in the cytoplasm and then in thhe mitochondria of the tumor cells. The inclusion of the PSs into mitochondriotropic liposomal formulations did not increase their uptake by cells but favored the interaction of the loaded molecules with the mitochondrial membranes. Finally, the results of dark and phototoxicity experiments showed that both compounds are nontoxic in the dark and are already efficient phototoxic agents for breast carcinoma cells at nM concentrations.

These results encourage further studies to explore the biological application of these new photosensitizers and could open new horizons in phototherapy. The possibility of entrapping the new PSs in lipidic vesicles further strengthens their application potential [46,75].

## Figures and Tables

**Table 1:** Emission data (fluorescence maxima, fluorescence quantum yields, lifetimes, radiative and non-radiative constants) and singlet oxygen production quantum yields of **C1** and **C2** in the three examined solvents.

Compound	Solvent	*λ*_max_ (nm) ^a^	*ϕ*_fl_ ^b^	*τ* (ns) ^c^	*k_r_ (* *s* ^−1^ *)*	*k_nr_ (* *s* ^−1^ *)*	*ϕ*_Δ_ ^d^
**C1**	TOL	664, 726	0.13	9.5	1.4 × 10^7^	9.1 × 10^7^	0.71
	DCM	660, 722	0.11	8.5	1.3 × 10^7^	1.0 × 10^8^	0.75
	DMSO	658, 722	0.14	10.5	1.3 × 10^7^	8.2 × 10^7^	- ^e^
**C2**	TOL	656, 718	0.027	3.7	7.3 × 10^6^	2.6 × 10^8^	- ^e^
	DCM	654, 718	0.019	3.3	5.8 × 10^6^	3.0 × 10^8^	0.73
	DMSO	652, 716	0.026	3.6	7.2 × 10^6^	2.7 × 10^8^	0.74

^a^ From corrected spectra. ^b^ Fluorescence quantum yields determined with reference to tetraphenylporphryin (TPP) in TOL (*ϕ*_fl_ = 0.11). ^c^ From single photon counting determinations, excitation at 560 nm. ^d^ Singlet oxygen production quantum yields measured (i) with direct ^1^O_2_ phosphorescence comparison with a standard (TPP in TOL and Rose Bengal bis(triethyl-ammonium) salt in DCM; see the Materials and Methods Section for details); (ii) with an indirect method that makes use of DPBF as singlet oxygen trap in DMSO (see the Materials and Methods Section for details). ^e^ Low solubility precluded the measurement.

**Table 2:** Physico-chemical properties of PS-loaded liposomes ^a^.

Liposome	DOPC/TPP3	Total Lipids/PS	R ± sd (nm)	PDI ± sd	ζ pot ± sd (mV)	EE%
DOPC/TPP3/**C1**	9.75:0.25	250:1	50.2 ± 0.2	0.114 ± 0.004	10.5 ± 0.4	40%
DOPC/TPP3/**C2**	9.75:0.25	250:1	63.0 ± 0.3	0.12 ± 0.03	8 ± 1	18%

^a^ In order to improve the EE%, additional sonication cycles with a tip sonicator have been performed before freeze–thawing. This strategy has been proved to be useful for other aromatic compounds, such as resveratrol [51,52], that usually form aggregates in aqueous solution, but in this case, it was not successful.

## Data Availability

The data supporting this article have been included as part of the Supplementary Materials.

## Supplementary Materials

The following supporting information can be downloaded at: https://www.mdpi.com/article/10.3390/molecules30132688/s1. Structural characterization of **C1** and **C2:** NMR characterization of **C1** (Figures S1–S4); NMR characterization of **C2** (Figures S5–S7); Crystal data and structure refinement for **C1** and **C2** (Table S1); Crystal structures of **C1** and **C2** and packing of **C1** along b-axis (Figure S8). Photophysical and theoretical characterization of **C1** and **C2**: Singlet oxygen quantum yield determination in DCM and DMSO (Figures S9–S10); Calculated absorption spectra of **C1** and **C2** (Figures S11–S15, Table S2); X,Y,Z coordinates of the ground state optimized systems with Ci symmetry (Pages S18–S20). Biological analyses: Absorption spectra of liposomes’ solutions at different concentrations (Figure S16); Flow cytometric analysis of intracellular uptake of **C1** and **C2** (Figure S17); LSCM analysis of intracellular localization of **C1** and **C2** in pharmaceutical solution (Figures S18–S19).

## Abbreviations

The following abbreviations are used in this manuscript:

PDT	Photodynamic therapy
PS	Photosensitizer
ROS	Reactive oxygen species
DNA	Deoxyribonucleic acid
MDA-MB-231	M D Anderson—Metastatic Breast—231
NMR	Nuclear magnetic resonance
COSY	Correlation spectroscopy
HSQC	Heteronuclear single quantum coherence
TOL	Toluene
DCM	Dichloromethane
DMSO	Dimethyl sulfoxide
DPBF	1,3-diphenylisobenzofuran
ZnPc	Zn-phthalocyanine
TPP	*meso* tetraphenylporphyrin
DFT	Density functional theory
TD-DFT	Time-dependent density functional theory
DOPC	1,2-dioleoyl-*sn*-glycero-3-phosphocholine
TPP3	Triphenylphosphonium bolaamphiphile
DMEM	Dulbecco’s Modified Eagle Medium
PBS	Phosphate-buffered saline
PDI	Polydispersity index
EE	Entrapment efficiency
CI	Cell Index
RTCA	Real-time cell analysis
LSCM	Laser scanning confocal microscopy
CCD	Charged coupled device
CCDC	Cambridge Crystallographic Data Center
MTS	3-(4,5-dimetiltiazol-2-il)-5-(3-metilfenil)-2-(4-metilfenil)-2H-tetrazolio
RPMI	Roswell Park Memorial Institute
