# Supplementary material for: Novel Azole-Modified Porphyrins for Mitochondria-Targeted Photodynamic Therapy
Source: Molecules. 2025 Jun 21;30(13):2688. doi: 10.3390/molecules30132688 (PMC12250747; doi:10.3390/molecules30132688)
Supplement: Supplementary file 1 [file molecules-30-02688-s001.zip › molecules-3650708-supplementary.pdf]

Supplementary Materials

for

Novel Azole-Modified Porphyrins for Mitochondria-Targeted Photodynamic Therapy

Sabarinathan Rangasamy,[1,2,*] Elisa Bandini,[1] Alessandro Venturini,[1] Giuseppina Bozzuto,[3] Sofia Migani,[3,4] Annarica Calcabrini,[3] Simona Sennato,[5] Caterina Zuffa,[6] Lucia Maini,[6] Anaïs Brion,[7] Frédéric Bolze,[7] Cecilia Bombelli,[8] Barbara Ventura[1,*]

1. *Institute for Organic Synthesis and Photoreactivity (ISOF), National Research Council of Italy (CNR), Via P. Gobetti 101, I-40129 Bologna, Italy.*
2. *Department of Chemistry, PSG College of Technology, Avinashi Rd, Peelamedu, Coimbatore, Tamil Nadu IN-641004, India.*
3. *National Centre for Drug Research and Evaluation, Italian National Institute of Health, Viale Regina Elena 299, I-00161 Rome, Italy.*
4. *School of Science and Technology, Chemistry Division, University of Camerino, via Madonna delle Carceri (ChIP), I-62032 Camerino, Italy*
5. *Institute for Complex Systems (ISC), National Research Council of Italy (CNR) and Physics Department, Sapienza University of Rome, Piazzale A. Moro 5, I-00185, Rome, Italy.*
6. *Department of Chemistry "Giacomo Ciamician", University of Bologna, Via F. Selmi 2, I-40126 Bologna, Italy.*
7. *Laboratory of Synthetic and Therapeutic Chemo-Biology, UMR 7199, University of Strasbourg/CNRS, Faculty of Pharmacy, Illkirch F-67401, Cedex, France.*
8. *Institute for Biological Systems (ISB), National Research Council of Italy (CNR), Secondary Office of Rome-Reaction Mechanisms c/o Department of Chemistry, Sapienza University of Rome, Piazzale A. Moro 5, I-00185 Rome, Italy.*

Table of contents

Structural characterization of **C1** and **C2** S2

- NMR characterization of **C1** (^{1}H, COSY, HSQC and ^{13}C NMR) S2
- NMR characterization of **C2** (^{1}H, HSQC and ^{13}C NMR) S6
- Crystal data and structure refinement for compounds **C1** and **C2** S9
- Crystal structures of **C1** and **C2** and packing of **C1** along b-axis S10

Photophysical and theoretical characterization of **C1** and **C2** S11

- Singlet oxygen quantum yield determination in DCM and DMSO S11
- Calculated absorption spectra of **C1** and **C2** S12
- X,Y,Z coordinates of the ground state optimized systems with C_i symmetry S18

Biological analyses S15

- Absorption spectra of liposomes' solutions at different concentrations S15
- Flow cytometric analysis of intracellular uptake of **C1** and **C2** S15
- LSCM analysis of intracellular localization of **C1** and **C2** in pharmaceutical solution S16

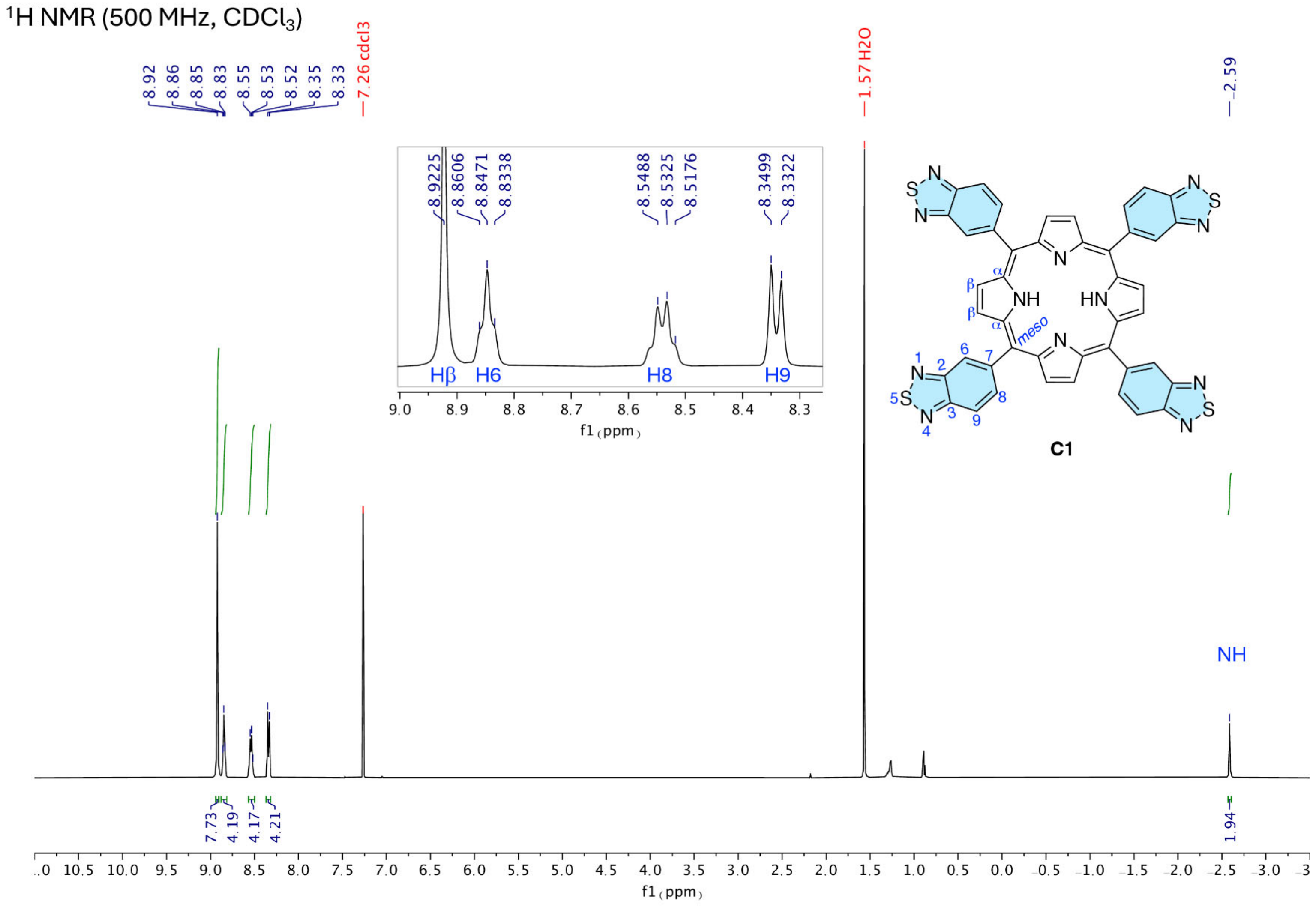

Figure S1. ^{1}H NMR spectrum (500 MHz, $CDCl_3$) of compound **C1** recorded at 25 °C.

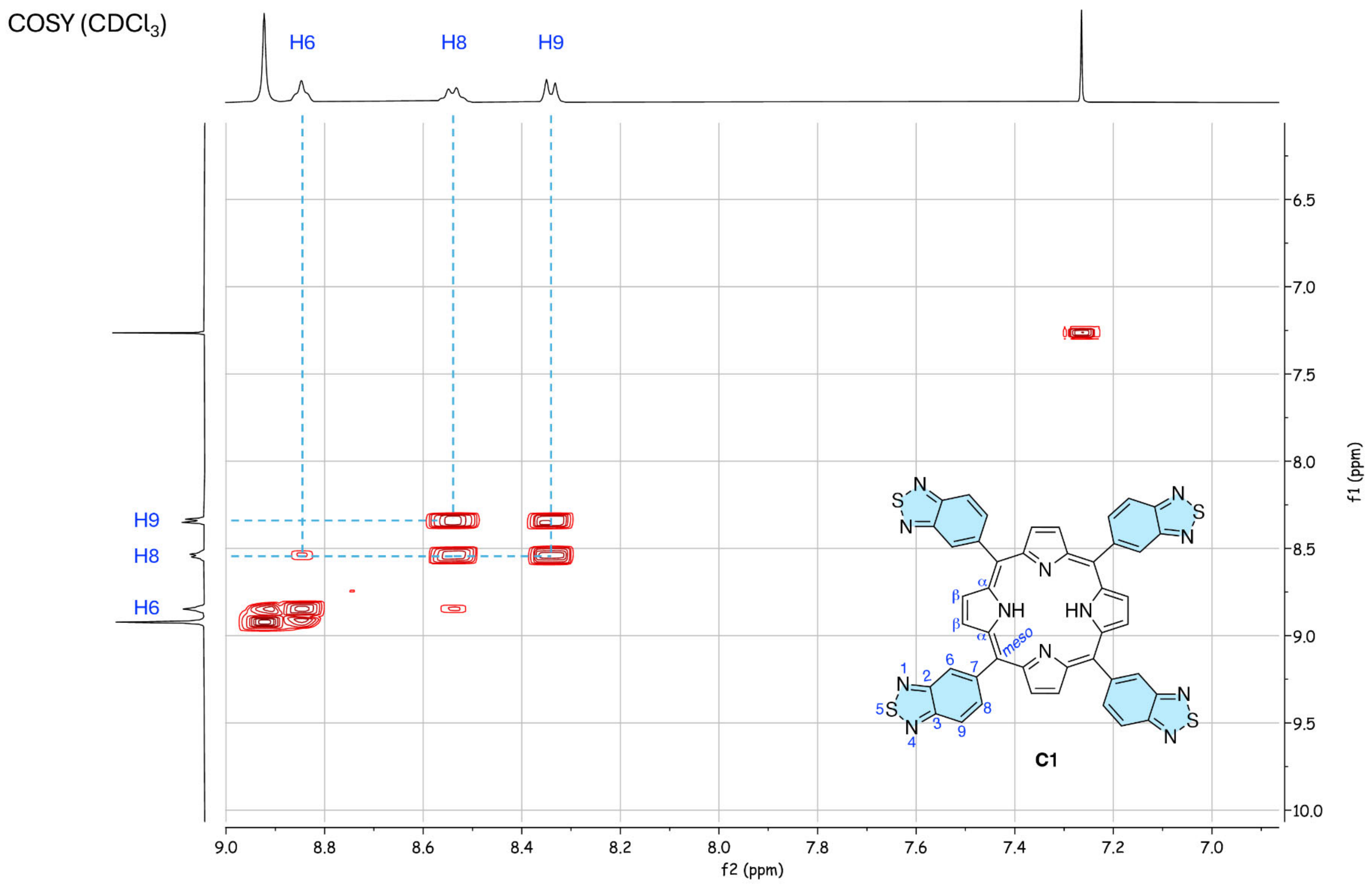

Figure S2. COSY NMR spectrum (500 MHz, $CDCl_3$) of compound **C1** recorded at 25 °C.

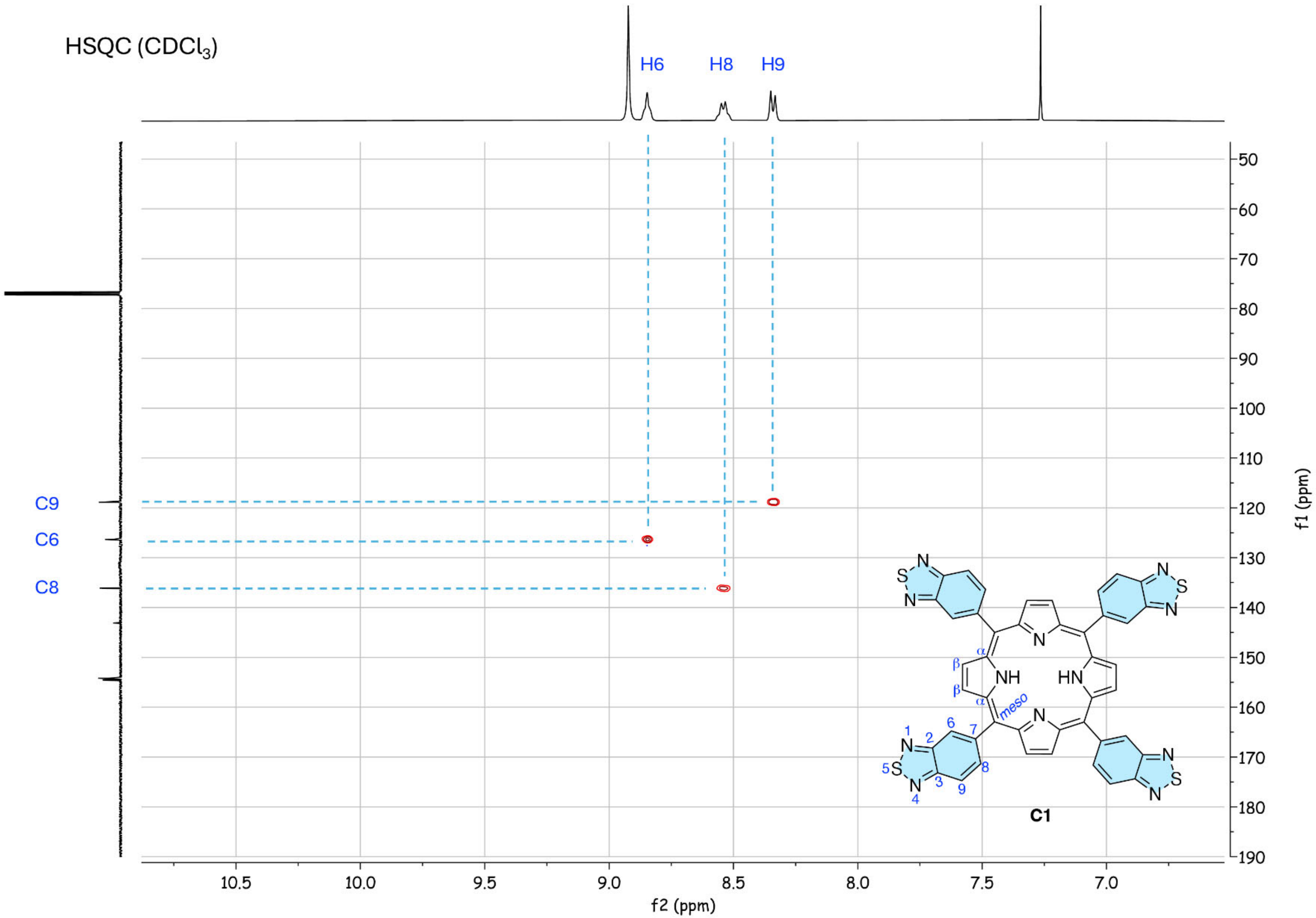

Figure S3. HSQC NMR spectrum (500 MHz, $CDCl_3$) of compound **C1** recorded at 25 °C.

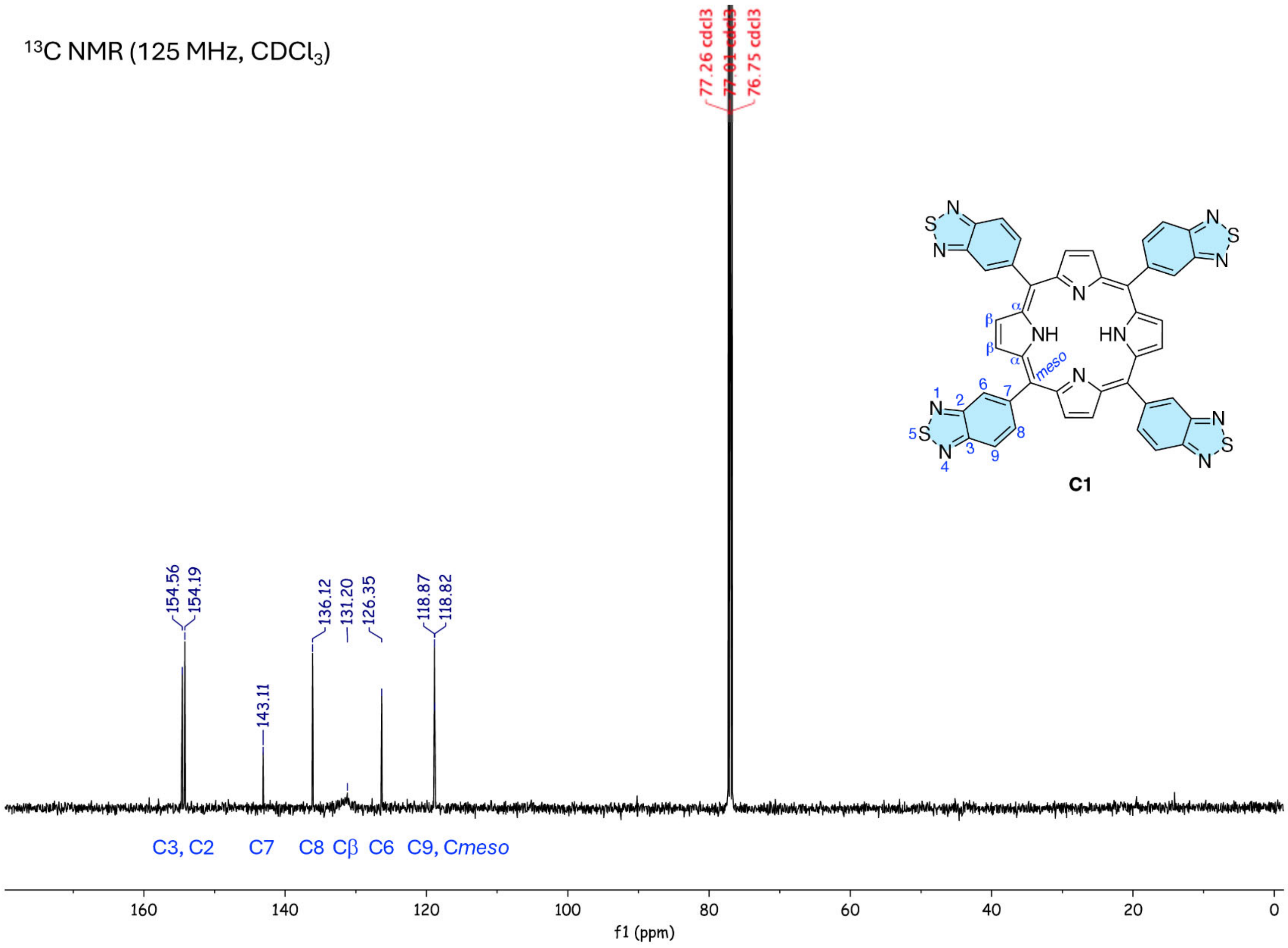

Figure S4. ^{13}C NMR spectrum (125 MHz, CDCl$_3$) of compound **C1** recorded at 25 °C.

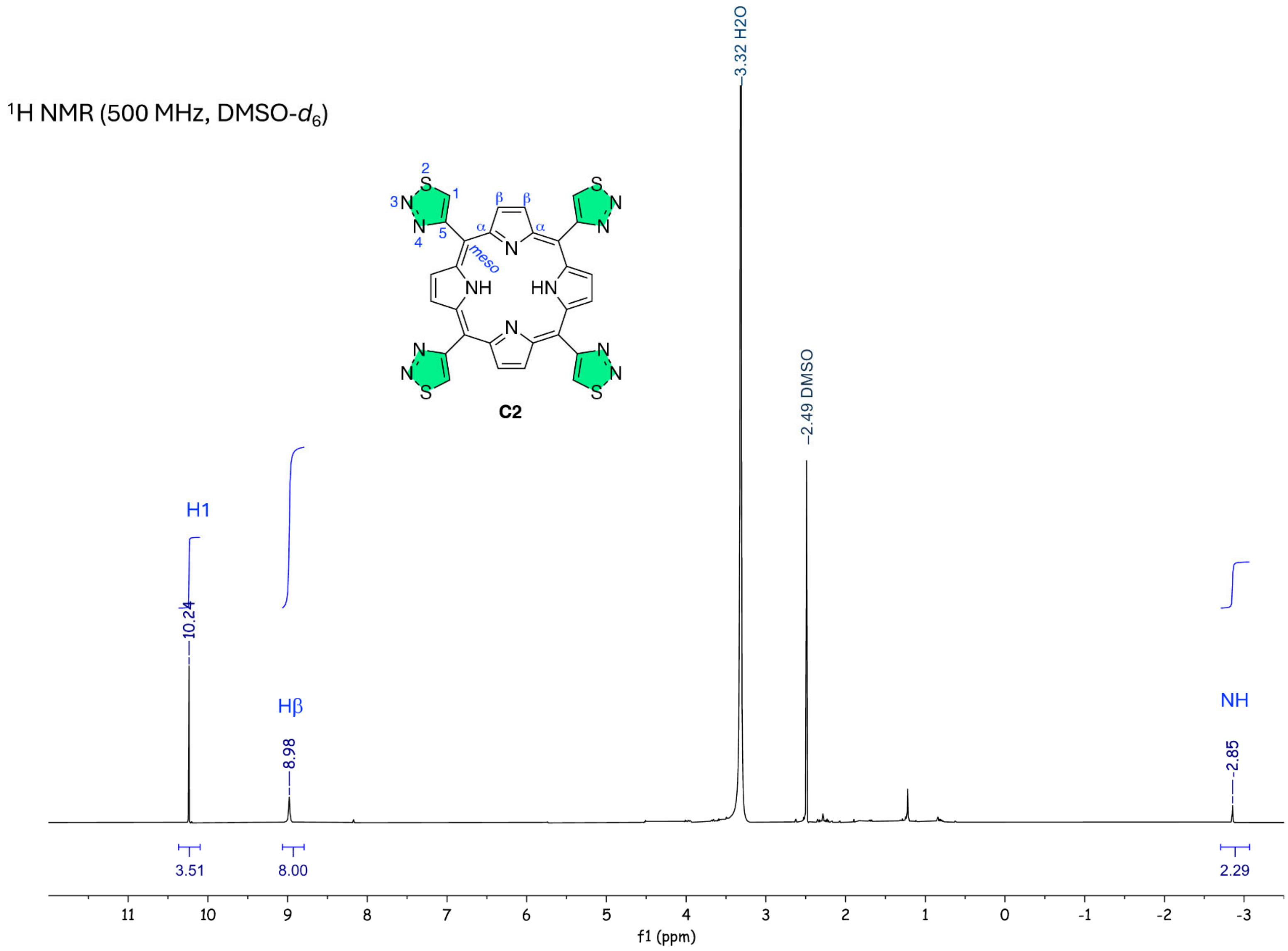

Figure S5. ^{1}H NMR spectrum (500 MHz, DMSO-d_6) of compound **C2** recorded at 25 °C.

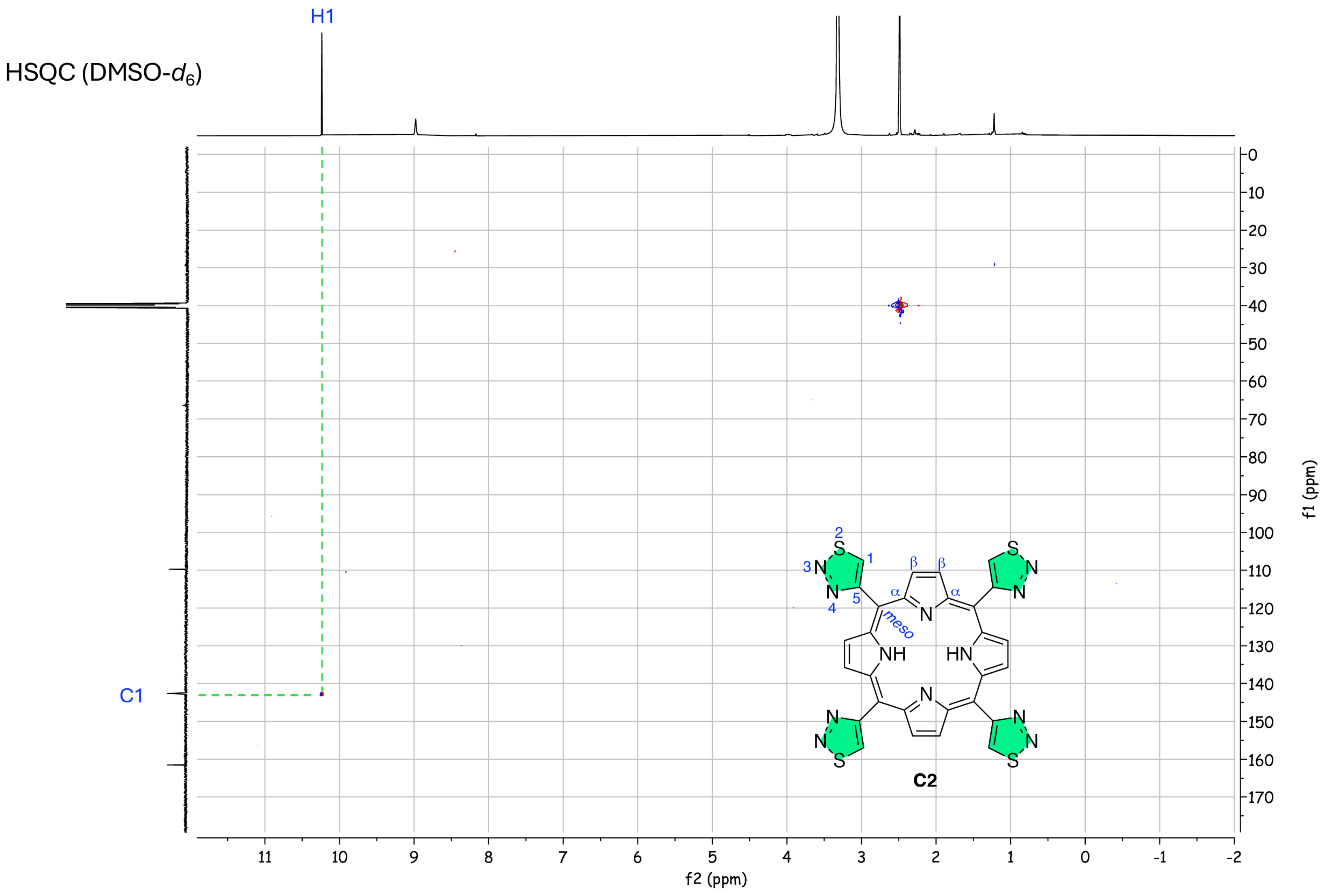

Figure S6. HSCQ NMR spectrum (500 MHz, DMSO-d_6) of compound **C2** recorded at 25 °C.

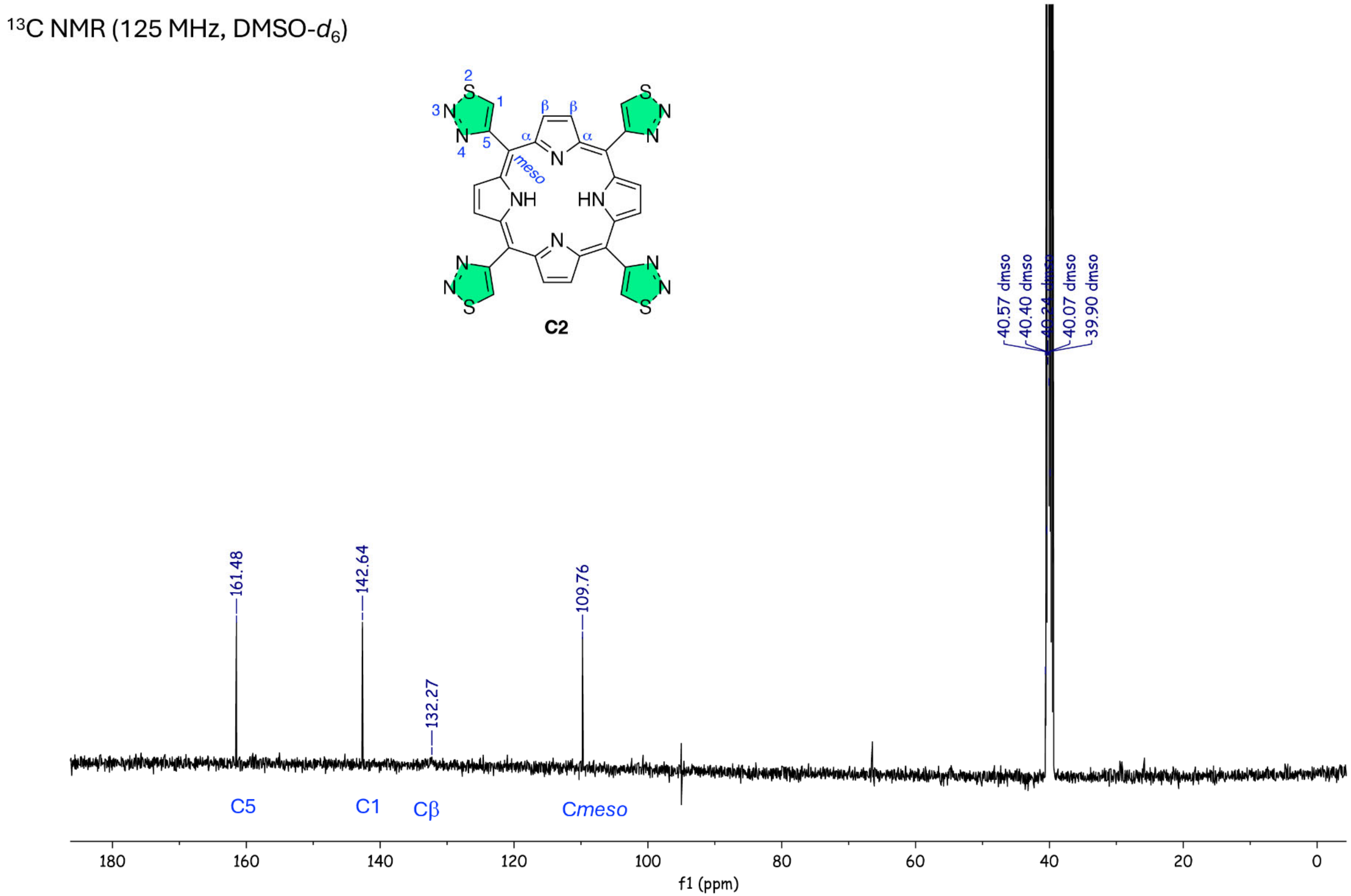

Figure S7. ^{13}C NMR spectrum (125 MHz, DMSO-d_6) of compound **C2** recorded at 25 °C.

Table S1. Crystal data and structure refinement for compounds **C1** and **C2**.

	**C1**	**C2**
Empirical formula	$C_{44}H_{20}N_{12}S_4$	$C_{28}H_{14}N_{12}S_4$
Formula weight (g mol^{-1})	844.96	646.75
T (K)	293	293
Wavelength (Å)	0.71073	0.71073
Crystal system	monoclinic	monoclinic
Space group	C2/c	P2/n
a (Å)	26.264(2)	14.8462(19)
b (Å)	7.0450(8)	5.9109(12)
c (Å)	24.5803(17)	15.8655(14)
a (°)	90	90
b (°)	99.677(6)	94.026(10)
g (°)	90	90
V (Å^3)	4483.5(7)	1388.8(4)
Z, Z'	4, 0.5	2, 0.5
ρ_{calc} (mg m^{-3})	1.252	1.547
μ (mm^{-1})	0.257	0.388
F(000)	1728	660
crystal size (mm)	0.524 x 0.079 x 0.023	0.476 x 0.087 x 0.028
θ range for data collection (◦)	3.363° to 29.103°	3.447° to 29.302°
reflections collected	10747	5883
Independent reflections	5083	3150
R_{int}	0.1188	0.1066
Completeness to theta = 25.000°	99.7%	99.8%
Refinement method	Full-matrix least-squares on F^2	Full-matrix least-squares on F^2
Tmax/Tmin	1.00000/0.76571	1.00000/0.04995
data/restraints/parameters	5083/0/278	3150/0/200
Goodness-of-fit on F^2	1.057	1.036
R1 [I > 2σ(I)]	0.1448	0.0997
*w*R2 (all data)	0.4274	0.2756

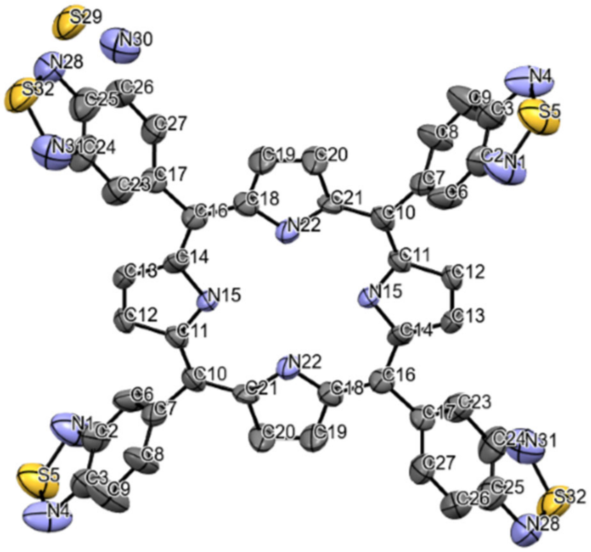

a)

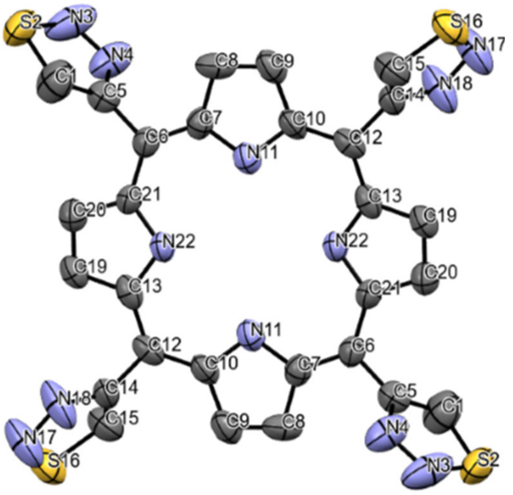

b)

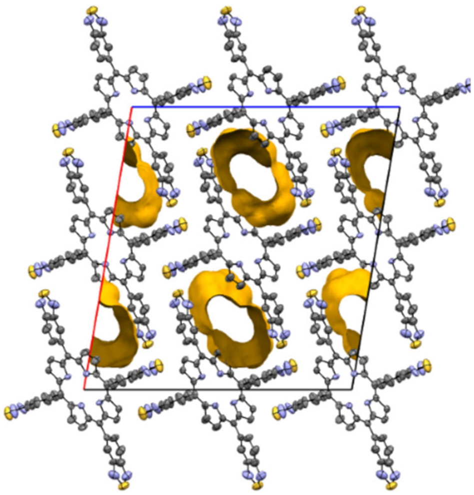
c)

Figure S8. Crystal structures showing the numbering of the atoms and ellipsoid at 50% of probability, in both cases the complete molecules are shown, while the asymmetric unit correspond to only half molecule. (a) **C1** showing the benzothiadiazole group disordered. The two positions were modelled with the N and S atoms at 0.5 occupancy in positions N30 and S29 or N31 and S32. The occupancy of 0.5 is determined by the presence of the inversion centre. (b) **C2**. In (c) the packing of **C1** viewed along b-axis is reported, highlighting the presence of voids. The residual electron density is too low to be ascribed to disordered solvent.

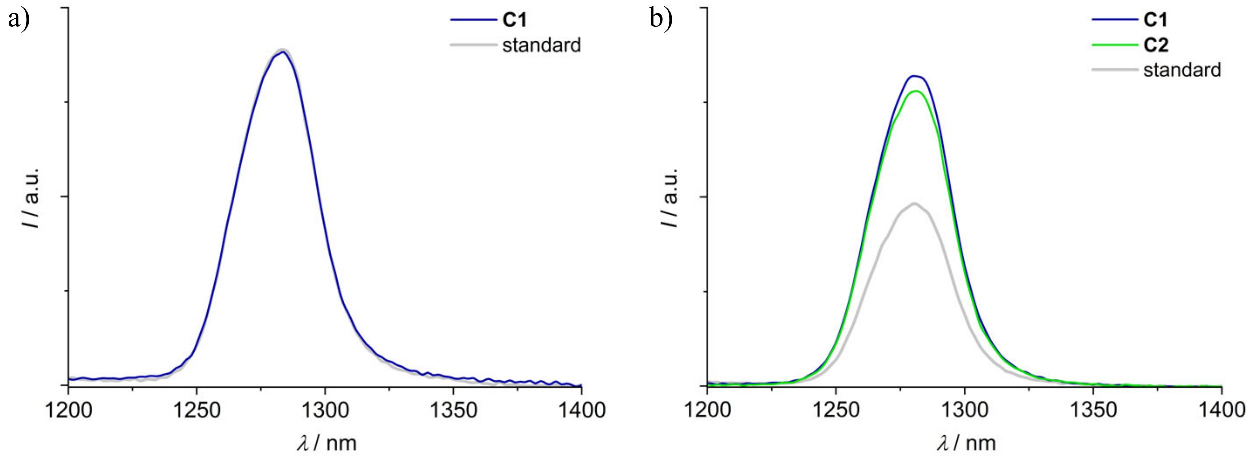

Figure S9. Singlet oxygen phosphorescence from optically matched solutions at 442 nm of a) **C1** and standard TPP in TOL and b) **C1**, **C2** and standard Rose Bengal bis(triethyl-ammonium) salt in DCM. A_{442} = 0.450.

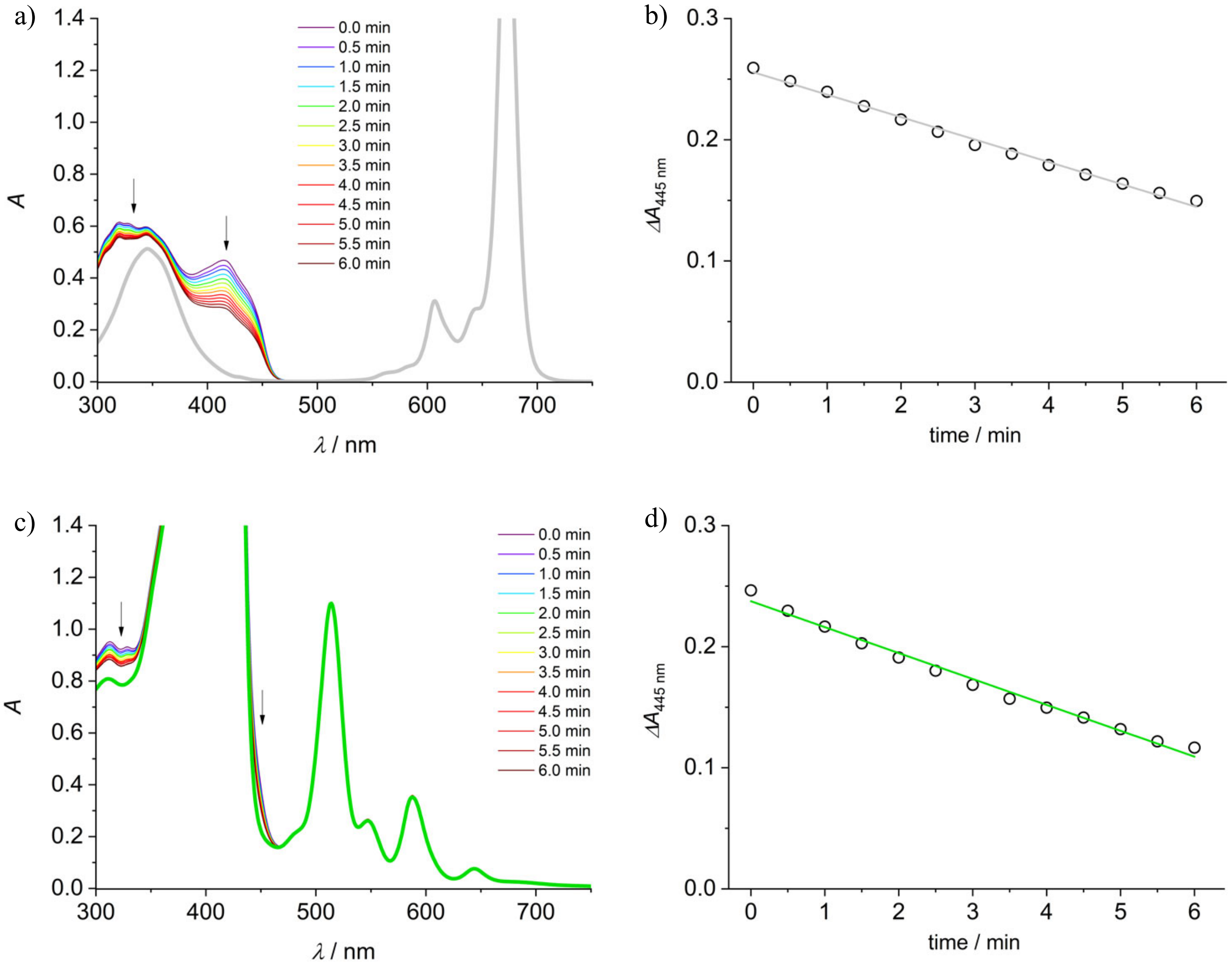

Figure S10. Absorption spectra of DMSO solutions containing (a) the standard ZnPc (grey) and DPBF or (c) compound **C2** and DPBF upon irradiation at 600 nm (0-8 min); the spectra of ZnPc and **C2** at the same concentration as in the mixtures are reported as thick lines in grey and green, respectively. (b) and (d): variation of DPBF absorbance at 445 nm as a function of the irradiation time for the cases (a) and (c), respectively; the linear fittings are reported as lines.

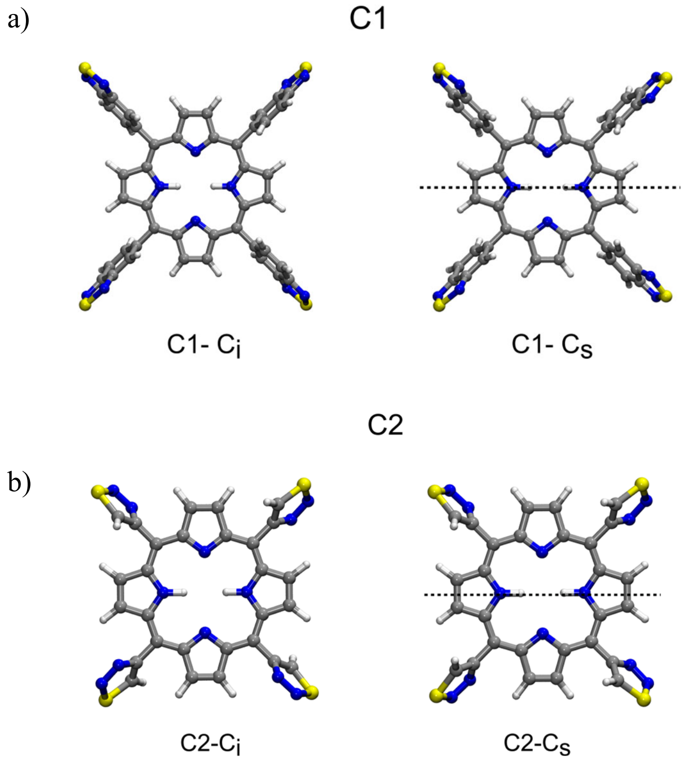

Figure S11. Studied isomers: a) **C1** and b) **C2** isomers. The dashed line in the Cs structures indicates the position of the perpendicular symmetry plane.

Table S2. Calculated values of the absorption spectra of the studied isomers (original values).

	Q_x band (λ, nm and *f*)				Soret band (λ, nm and *f*)			
**C1-C$_i$**	601.7	0.006	549.1	0.046	385.4	1.965	379.9	2.227
**C1-C$_s$**	600.2	0.015	547.5	0.028	386.3	1.949	379.9	2.246
**C2-C$_i$**	601.3	0.002	546.0	0.011	383.1	1.609	374.7	1.923
**C2-C$_s$**	599.8	0.002	546.6	0.010	383.1	1.608	374.0	1.920

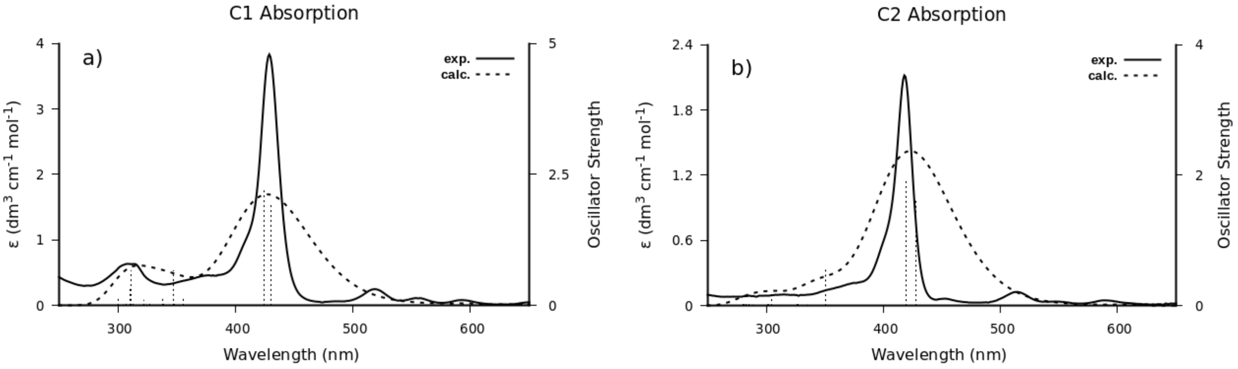

Figure S12. Absorption spectra of the **C1-C$_i$** and **C2-C$_i$** isomers (a and b, respectively). The λ values of the theoretical calculations are red-shifted by 45 nm to have a better overlap with the experimental Soret bands.

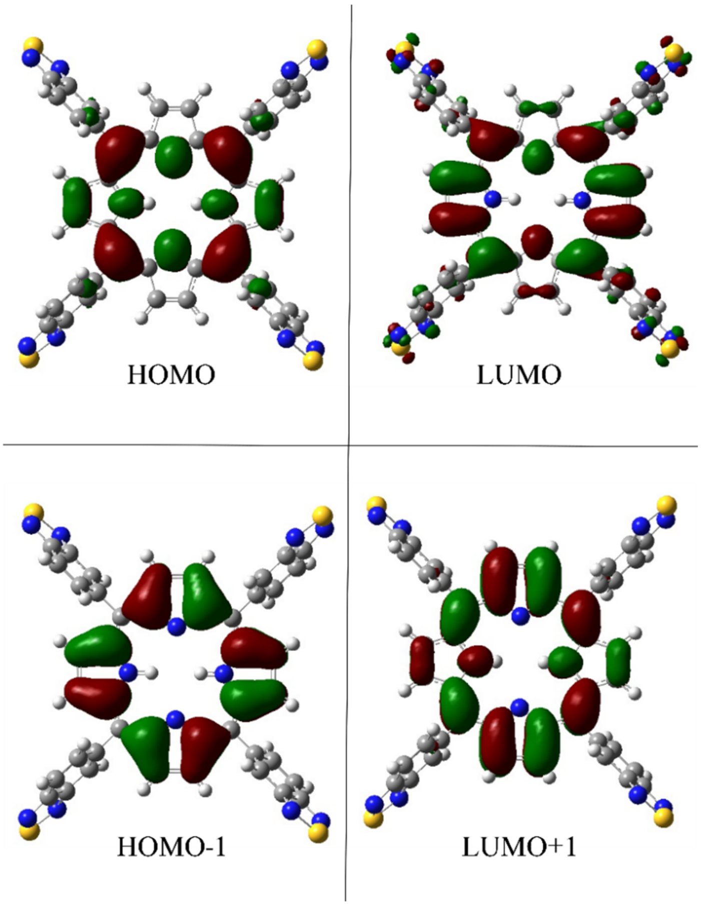

Figure S13. Orbitals involved in the Q and Soret transitions. **C1-C$_i$** structure.

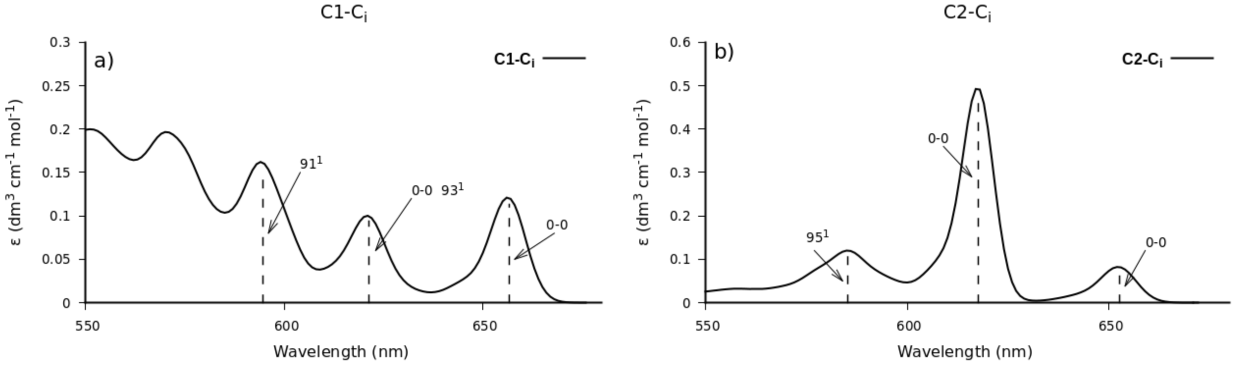

Figure S14. FCHT spectra of the two more stable structures **C1-C$_i$** (a) and **C2-C$_i$** (b). The spectra are dominated by the 0-0 transitions (i.e. from ground state to point zero energy of the excited state) and the other peaks are due to transitions to states with excited vibrations and are labelled as n^x, where n indicates the excited normal mode and x its quantum number.

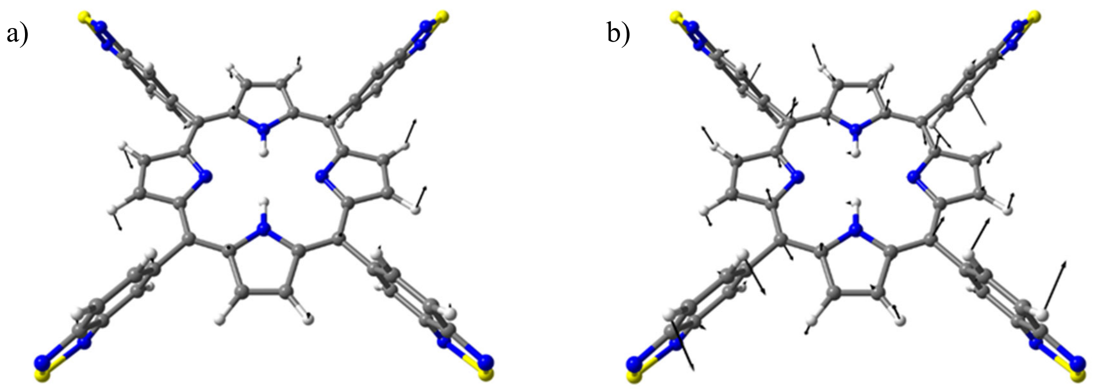

Figure S15. Illustration, through the arrows, of the two movements that concern the normal modes 91[1] (a) and 93[1] (b) of **C1-C$_i$**.

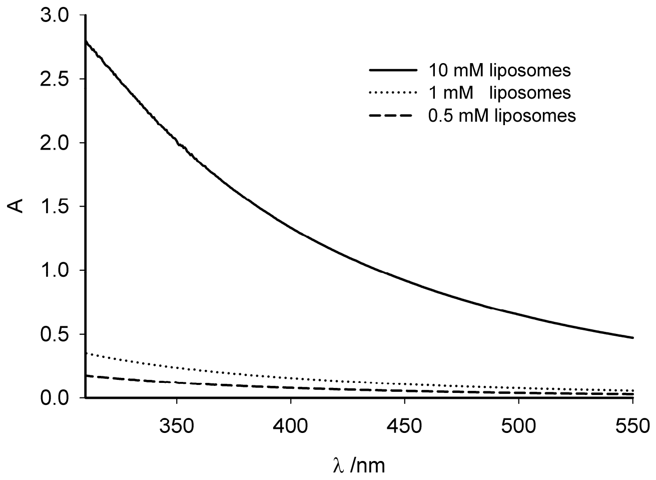

Figure S16. Absorption spectra of liposomes' solutions at different concentrations.

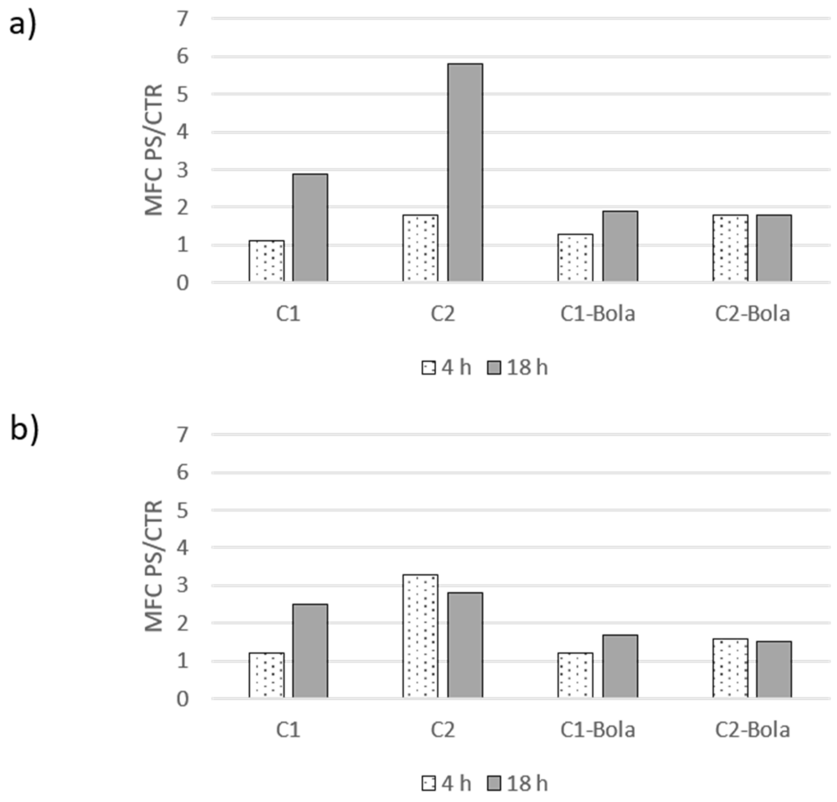

Figure S17. Flow cytometric analysis of MDA-MB-231 intracellular uptake of **C1** and **C2** administered alone or vehicled by mitochondriotropic liposomes (DOPC+TTP3_Bola). Photosensitizers (PS) were prepared in DMSO (a) or in pharmaceutical solution F (40% ethanol and 60% propylene glycol) (b). The ratios between the mean fluorescence channel (MFC) derived from cells incubated with the photosensitizers and that of untreated cells (CTR, incubation with vehicle alone) were calculated to quantify the increase of fluorescence emissions of treated *vs* untreated samples (showing autofluorescence signal).

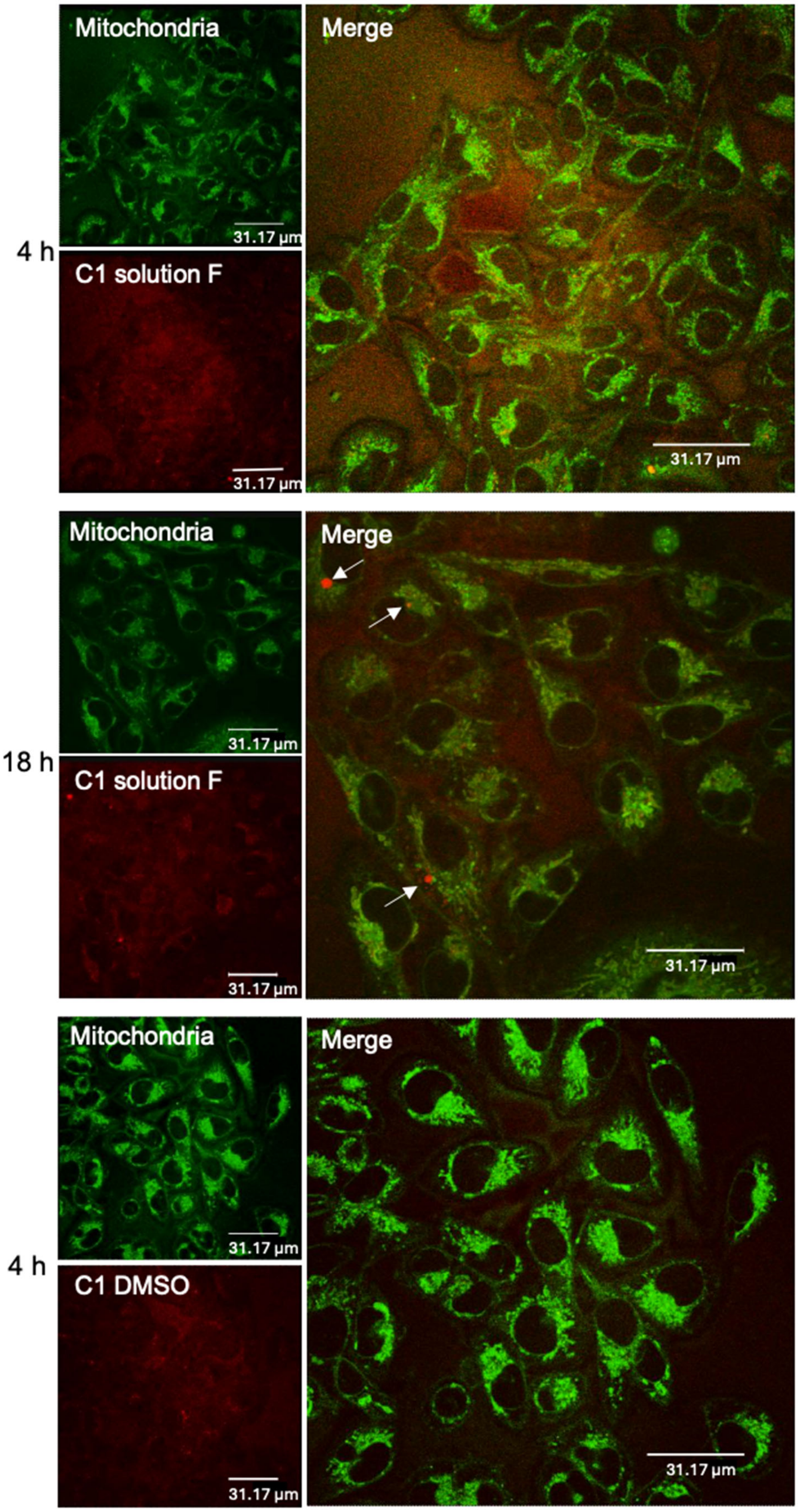

Figure S18. Analysis by LSCM of the intracellular localization of **C1** administered in pharmaceutical solution F (40% ethanol and 60% propylene glycol) and DMSO in MDA-MB-231 cells. Optical sections.

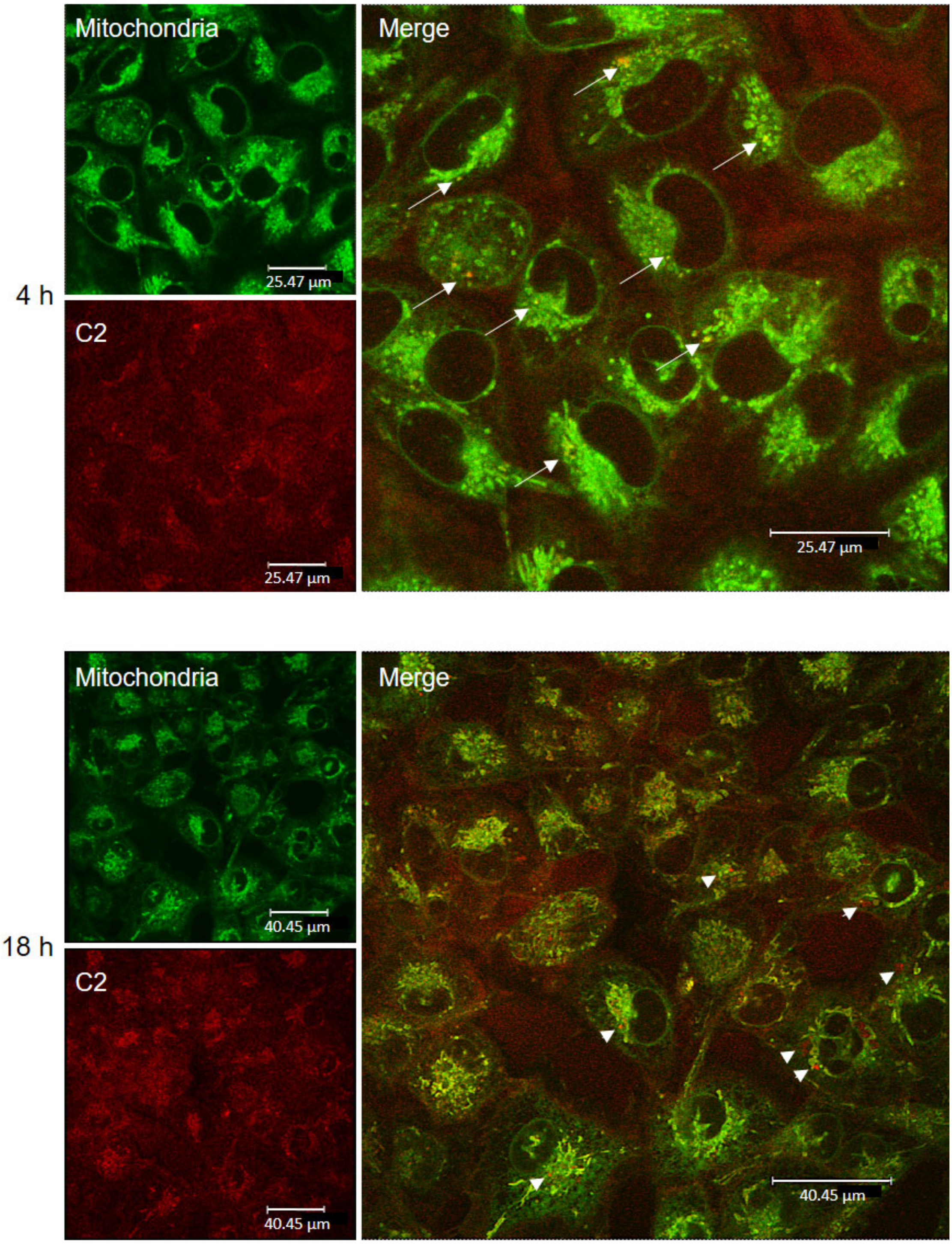

Figure S19. Analysis by LSCM of the intracellular localization of **C2** administered in pharmaceutical solution F (40% ethanol and 60% propylene glycol) in MDA-MB-231 cells. Optical sections.

X,Y,Z coordinates of the ground state optimized systems with C_i symmetry.

C1-C_i

$\Delta\Delta G$ = -3936.07812 (a.u.)

S	4.00146300	0.98271300	-8.60208900
S	-8.29557100	-0.99948000	-4.60236400
N	-0.63169300	-0.00425000	-1.93590200
N	-1.98159500	0.22804500	0.64546100
H	-1.01564100	0.16576400	0.33057900
C	-1.90991900	0.08802800	-2.38155300
C	-1.96057600	0.05632400	-3.84064000
H	-2.85872100	0.12116800	-4.45239300
C	2.21851600	-0.30207000	-4.40997600
C	-0.67969100	-0.06649900	-4.25783100
H	-0.31396900	-0.13098500	-5.28118700
C	0.13869000	-0.09736100	-3.04889400
C	-2.37299400	0.26538800	1.95529700
C	1.53995200	-0.21607900	-3.07888100
C	-3.07018800	0.26127100	-0.18154800
C	-3.05985700	0.20773800	-1.58019700
C	-4.39268900	0.29068300	-2.25540800
C	-3.80602000	0.33444000	1.95974200
H	-4.42427200	0.36593100	2.85497800
C	2.36404800	0.81429200	-5.19114800
H	1.99653200	1.79039000	-4.86789800
C	2.71434600	-1.58156100	-4.85441700
H	2.57210600	-2.44262700	-4.19534800
C	-4.23013900	0.33173300	0.65992600
H	-5.25752000	0.36047400	0.30167800
C	-5.06373600	1.56631500	-2.31123700
H	-4.56614300	2.42684100	-1.85523200
N	4.08360800	-0.55728500	-8.09278800
C	-4.96319600	-0.82452900	-2.81101900
H	-4.46958200	-1.79785200	-2.77495100
C	3.01220400	0.68425600	-6.45549600
C	3.50408200	-0.60016300	-6.89278100
N	-8.07206300	0.53795900	-4.12871700
N	3.23639300	1.65800500	-7.33882800
C	-6.27963000	1.72792700	-2.91222600
H	-6.77828000	2.69810900	-2.95354900
C	3.34078300	-1.74595200	-6.05708100
H	3.70925000	-2.71895200	-6.38742200
C	-6.23279700	-0.69729500	-3.44941500
C	-6.89661000	0.58311600	-3.50107800
N	-6.92813300	-1.67047700	-4.03944100
S	-4.00146300	-0.98271300	8.60208900
S	8.29557100	0.99948000	4.60236400
N	0.63169300	0.00425000	1.93590100
N	1.98159500	-0.22804500	-0.64546100
H	1.01564100	-0.16576400	-0.33057900
C	1.90991900	-0.08802800	2.38155300
C	1.96057600	-0.05632400	3.84064000
H	2.85872100	-0.12116800	4.45239300
C	-2.21851600	0.30207000	4.40997600
C	0.67969100	0.06649900	4.25783100

H	0.31396900	0.13098500	5.28118700
C	-0.13869000	0.09736100	3.04889400
C	2.37299400	-0.26538800	-1.95529700
C	-1.53995200	0.21607900	3.07888100
C	3.07018800	-0.26127100	0.18154800
C	3.05985700	-0.20773800	1.58019700
C	4.39268900	-0.29068300	2.25540800
C	3.80602000	-0.33444000	-1.95974200
H	4.42427200	-0.36593100	-2.85497800
C	-2.36404800	-0.81429300	5.19114800
H	-1.99653200	-1.79039000	4.86789800
C	-2.71434600	1.58156100	4.85441700
H	-2.57210600	2.44262700	4.19534800
C	4.23013900	-0.33173300	-0.65992600
H	5.25752000	-0.36047400	-0.30167800
C	5.06373600	-1.56631500	2.31123700
H	4.56614300	-2.42684100	1.85523200
N	-4.08360800	0.55728500	8.09278800
C	4.96319600	0.82452900	2.81101900
H	4.46958200	1.79785200	2.77495100
C	-3.01220400	-0.68425600	6.45549600
C	-3.50408200	0.60016300	6.89278100
N	8.07206300	-0.53795900	4.12871700
N	-3.23639300	-1.65800500	7.33882800
C	6.27963000	-1.72792700	2.91222600
H	6.77828000	-2.69810900	2.95354900
C	-3.34078300	1.74595100	6.05708100
H	-3.70925000	2.71895200	6.38742200
C	6.23279700	0.69729500	3.44941500
C	6.89661000	-0.58311600	3.50107800
N	6.92813300	1.67047700	4.03944100

C2-C$_i$

ΔΔG = -3322.28777 (a.u.)

S	0.54447100	-2.89704600	6.76136600
S	0.52670500	-6.79270100	-2.83520800
N	-0.08258500	0.84111300	1.92440000
H	-0.11202300	0.43207700	0.99234600
N	-0.01757500	-1.86257400	0.81988100
C	-0.05056800	0.12451600	3.08808300
C	0.01517000	1.07363000	4.16152800
H	0.05838800	0.81879000	5.21798000
C	-0.02423800	-3.04466200	0.15595700
N	-1.10775900	-5.39809300	-1.62649500
C	-0.09620400	-3.63201900	2.32368800
H	-0.15391800	-4.15836100	3.27466800
C	-0.05301600	-2.18348200	2.13673700
C	-0.05074400	-1.27059300	3.20778000
C	-0.06841200	-4.17042600	1.08408500
H	-0.09046300	-5.22499600	0.81591600
C	-0.03473500	2.18162800	2.18692500
C	-0.02638600	-1.80652500	4.59229900
C	-0.00238400	-4.59966500	-1.77257500
C	0.02358300	2.32507600	3.61383700
H	0.08509400	3.27313900	4.14383200
N	-1.07541900	-1.58369400	5.44718500

C	0.00727100	-3.21268800	-1.24017200
N	-0.99390500	-6.56315900	-2.12110200
C	0.98863000	-2.53238400	5.17112900
H	1.93330000	-2.83458400	4.71985400
N	-0.93491500	-2.08334200	6.60729300
C	1.02687000	-5.22743700	-2.43422400
H	2.01063800	-4.82396700	-2.67288100
S	-0.54447100	2.89704600	-6.76136600
S	-0.52670500	6.79270100	2.83520800
N	0.08258500	-0.84111300	-1.92440000
H	0.11202300	-0.43207700	-0.99234600
N	0.01757500	1.86257400	-0.81988100
C	0.05056800	-0.12451600	-3.08808300
C	-0.01517000	-1.07363000	-4.16152800
H	-0.05838800	-0.81879000	-5.21798000
C	0.02423800	3.04466200	-0.15595700
N	1.10775900	5.39809300	1.62649500
C	0.09620400	3.63201900	-2.32368800
H	0.15391800	4.15836100	-3.27466800
C	0.05301600	2.18348200	-2.13673700
C	0.05074400	1.27059300	-3.20778000
C	0.06841200	4.17042600	-1.08408500
H	0.09046300	5.22499600	-0.81591600
C	0.03473500	-2.18162800	-2.18692500
C	0.02638600	1.80652500	-4.59229900
C	0.00238400	4.59966500	1.77257500
C	-0.02358300	-2.32507600	-3.61383700
H	-0.08509400	-3.27313900	-4.14383200
N	1.07541900	1.58369400	-5.44718500
C	-0.00727100	3.21268800	1.24017200
N	0.99390500	6.56315900	2.12110200
C	-0.98863000	2.53238400	-5.17112900
H	-1.93330000	2.83458400	-4.71985400
N	0.93491500	2.08334200	-6.60729300
C	-1.02687000	5.22743700	2.43422400
H	-2.01063800	4.82396700	2.67288100
